# Cerebral Microbleeds: A Review of Clinical, Genetic, and Neuroimaging Associations

**DOI:** 10.3389/fneur.2013.00205

**Published:** 2014-01-06

**Authors:** Paul A. Yates, Victor L. Villemagne, Kathryn A. Ellis, Patricia M. Desmond, Colin L. Masters, Christopher C. Rowe

**Affiliations:** ^1^Department of Nuclear Medicine and Centre for PET, Austin Health, Heidelberg, VIC, Australia; ^2^Department of Medicine, The University of Melbourne, Parkville, VIC, Australia; ^3^Florey Institute of Neuroscience and Mental Health, University of Melbourne, Parkville, VIC, Australia; ^4^Department of Radiology, Royal Melbourne Hospital, Parkville, VIC, Australia

**Keywords:** microbleeds, intracerebral hemorrhage, stroke, cerebral amyloid angiopathy, Alzheimer’s disease, MRI imaging, positron-emission tomography, amyloid imaging

## Abstract

Cerebral microbleeds (microbleeds) are small, punctuate hypointense lesions seen in T2* Gradient-Recall Echo (GRE) and Susceptibility-Weighted (SWI) Magnetic Resonance Imaging (MRI) sequences, corresponding to areas of hemosiderin breakdown products from prior microscopic hemorrhages. They occur in the setting of impaired small vessel integrity, commonly due to either hypertensive vasculopathy or cerebral amyloid angiopathy. Microbleeds are more prevalent in individuals with Alzheimer’s disease (AD) dementia and in those with both ischemic and hemorrhagic stroke. However they are also found in asymptomatic individuals, with increasing prevalence with age, particularly in carriers of the Apolipoprotein (APOE) ε4 allele. Other neuroimaging findings that have been linked with microbleeds include lacunar infarcts and white matter hyperintensities on MRI, and increased cerebral β-amyloid burden using ^11^C-PiB Positron Emission Tomography. The presence of microbleeds has been suggested to confer increased risk of incident intracerebral hemorrhage – particularly in the setting of anticoagulation – and of complications of immunotherapy for AD. Prospective data regarding the natural history and sequelae of microbleeds are currently limited, however there is a growing evidence base that will serve to inform clinical decision-making in the future.

## Introduction

Symptomatic Intracerebral Hemorrhage (ICH) affects 30–40 per 10,000 annually (1), and can have devastating clinical outcomes (2). Well-known modifiable risk factors for ICH include hypertension (3), smoking (4), alcohol (5, 6), and diabetes (7). In addition, recent developments in neuroimaging have led to a greater understanding of pathophysiology and risk of ICH (8).

Presence of hypertensive arteriolosclerosis and cerebral amyloid angiopathy (CAA) are contributory in an estimated 78–88% of primary ICH (9). In both of these conditions, prior to emergence of symptomatic ICH, there may be evidence of smaller, possibly subclinical hemorrhages, reflective of underlying vascular fragility. These lesions, termed cerebral “microbleeds” (microbleeds) may be an indicator of increased risk for future macroscopic hemorrhage (10, 11).

As well as being associated with ICH, microbleeds are associated with ischemic stroke (IS) (12), Alzheimer’s Disease (AD) (13), and AD immunotherapy (14, 15), and they are seen with an age-dependent higher prevalence in cognitively normal elderly (16, 17). They are also seen secondary to trauma, inflammatory conditions, and several genetic disorders (18, 19).

Previously considered to be clinically silent (20–22), an increasing number of studies have linked presence of microbleeds and cognitive decline (23, 24), in addition to being a putative marker of future stroke risk (10, 25, 26).

This review recapitulates recent clinical and neuroimaging literature regarding cerebral microbleeds, in particular addressing their associated risk factors and prognostic implications.

### Histopathology and nomenclature

The terms, “cerebral microbleeds,” or “microhemorrhages,” refer to small, round, or ovoid hypointensities, of <10 mm in diameter, evident on T2* Gradient-Recall Echo (GRE) or Susceptibility-Weighted (SWI) MRI sequences. These sequences provide high contrast between brain parenchyma and paramagnetic material, such as deoxyhemoglobin, superparamagnetic hemosiderin, and diamagnetic calcium (27, 28), and are capable of detecting bleeding from vessels as small as 200 μm in diameter (29). An associated finding on these magnetic resonance imaging (MRI) sequences is superficial siderosis (SS), presence of residual leptomeningeal hemosiderin deposits after small vessel rupture within the subarachnoid space (30).

The terminology distinguishes “microbleeds” seen on MRI from small lesions visible under light microscopy at post-mortem [also termed “mini-bleeds” (31)].

The first histopathological correlations with microbleeds were published by Tanaka et al. (29) and Fazekas et al. (32) each noting that sites of hypointensities seen on MRI corresponded to areas of hemosiderin, deposited around arteriosclerotic vessels (29, 32). More recently, Shoamanesh and colleagues reviewed the histopathology of 18 patients from five published studies: six with dementia, seven ICH, one IS, and four with other pathologies. Microbleeds on MRI were associated with evidence in prior bleeding in 81% (e.g., hemosiderin-laden macrophages or old hematoma). Other infrequent findings included pseudocalcification, a microaneurysm, and a distended dissected vessel. Other non-specific findings were seen in 13% of cases. Associated vascular pathology seen was most commonly lipohyalinosis or vascular β-amyloid deposition, the latter seen predominantly in individuals with dementia (33).

Evidence from both clinico-pathological correlations and large epidemiological studies also support differing patterns of distribution of microbleeds according to their etiology. MB in deep subcortical or infratentorial areas are usually associated with the presence of hypertensive disease or vascular risk factors (VRF) (16, 34), with lipohyalinosis being the predominant finding at post-mortem (33). Hemorrhages in a lobar, cortico-subcortical distribution are associated with Apolipoprotein E (APOE) ε2 (35) and APOE ε4 carrier status (16, 34), β-amyloid burden on ^11^C-PiB positron emission tomography (PET) (36), and evidence of CAA at post-mortem (32). In the setting of trauma, microbleeds have been reported more frequently in mid-subcortical cerebrum, above the corpus callosum, whereas non-traumatic microbleeds were found in lateral subcortical areas, basal ganglia, and thalamus (37). Microbleeds in the cerebellum have been associated with both presence of CAA and with arteriosclerotic disease (38).

In individuals presenting with symptomatic ICH, criteria to support diagnosis of CAA have been proposed (1) and validated with histopathology, known as the Boston Criteria (39), outlined in Table 1. Inclusion of microbleeds (40) and SS (41) have been suggested to improve the sensitivity of the Boston Criteria for detection of CAA, particularly with lesions detected in asymptomatic individuals.

**Table 1:** **Boston criteria for cerebral amyloid. angiopathy^a^**.

**DEFINITE CAA**
Full post-mortem examination demonstrating
Lobar, cortical, or cortico-subcortical hemorrhage
Severe CAA with vasculopathy^b^
Absence of other diagnostic lesion
**PROBABLE CAA WITH SUPPORTING PATHOLOGY**
Clinical data and pathologic tissue (evacuated hematoma or cortical biopsy) demonstrating
Lobar, cortical, or cortico-subcortical hemorrhage
Some degree of CAA in specimen
Absence of other diagnostic lesion
**PROBABLE CAA**
Clinical data and MRI or CT demonstrating
Multiple hemorrhages restricted to lobar, cortical, or cortico-subcortical regions (cerebellar hemorrhage allowed)
Age ≥ 55 years
Absence of other cause of hemorrhage^c^
**POSSIBLE CAA**
Clinical data and MRI or CT demonstrating:
Single lobar, cortical, or cortico-subcortical hemorrhage
Age ≥ 55 years
Absence of other cause of hemorrhage^c^

*^a^Criteria established by the Boston Cerebral Amyloid Angiopathy Group: Steven M. Greenberg, MD, Ph.D., Daniel S. Kanter, MD, Carlos S. Kase, MD, and Michael S. Pessin, MD*.

*^b^As defined in Ref. (42)*.

^c^Other causes of intracerebral hemorrhage: excessive warfarin (INR 3.0); antecedent head trauma or ischemic stroke; CNS tumor, vascular malformation, or vasculitis; and blood dyscrasia or coagulopathy. (INR 3.0 or other non-specific laboratory abnormalities permitted for diagnosis of possible CAA.)

In patients with probable CAA, lobar microbleeds occur more frequently in posterior structures (43). This corresponds to the distribution of CAA-laden vessels described at post-mortem (44). A similar pattern of distribution has been observed in AD patients, supporting that that CAA also underlies a majority of these lesions in AD (13). Other studies have reported discordant findings, or variability according to clinical groups or definition of anatomical landmarks. In one study of community-dwelling elderly, lobar microbleeds were most prevalent in posterior temporal and parietal, but not occipital lobes, while the large, population-based Age, Gene/Environment Susceptibility study (AGES) showed no regional predominance at all (17). One study of patients with subcortical vascular dementia (VaD), with high prevalence of VRF and Lacunar Infarction (LI) (in whom one might expect to find deep MB) the majority of lesions detected were actually *lobar* microbleeds (45) However, this study did not include a β-amyloid biomarker [e.g., cerebrospinal fluid (CSF) or PET imaging] and so it is possible that many of these patients may have had mixed pathologies (e.g., LI with concomitant AD-pathology).

## Genetic Associations of Microbleeds

Genetic factors associated with microbleeds include polymorphisms linked with sporadic microbleeds and less common mutations seen with familial conditions.

The most common gene polymorphism associated with sporadic microbleeds is the Apolipoprotein E (APOE) gene on chromosome 19. The APOE ε2 and ε4 alleles have each been independently associated with lobar microbleeds (16, 35), APOE ε4 associated with greater vascular Aβ deposition, with loss of smooth muscle and vessel wall thickening (46–48), whereas ε2 with fibrinoid necrosis (49). In their meta-analysis of over 7000 subjects, Maxwell and colleagues found that ε4 was also associated with deep microbleeds, but also that there was no increase in odds of microbleeds in ε2 compared with ε3 (50).

In addition, genome-wide association studies (GWAS) have identified polymorphisms associated with more severe CAA include neprilysin (a proteolytic enzyme responsible for Aβ catabolism (51)) and single-nucleotide polymorphism rs6656401 within the Complement Receptor-1 gene (52). It could be inferred that these also represent higher risk of microbleeds, although this remains to be demonstrated.

Mutations associated with microbleeds in familial conditions include NOTCH-3 in Cerebral Autosomal Dominant Arteriopathy with Subcortical Infarcts and Leukoencephalopathy (CADASIL) (53), APP E693Q and D694N in Dutch-type (40, 54) or Iowa-type (55) CAA, and APP and presenilin mutations in familial AD (56, 57).

## Microbleeds and Neuroimaging

### MRI: On sequences and findings

The most commonly used sequences to demonstrate microbleeds are T2* Gradient-Recall Echo (T2*GRE) and Susceptibility-Weighted (SWI) MRI. These provide high contrast between brain parenchyma and highly paramagnetic material, such as deoxyhemoglobin, superparamagnetic hemosiderin, and diamagnetic calcium (27, 28), and are sensitive to rupture of blood vessels as small as 200 μm in diameter (29).

The choice of sequence and imaging parameters – such as echo time, field strength, and slice thickness – affects the size, clarity, and number of lesions identified (58, 59). This represents a source of heterogeneity between studies, and as such, can limit direct comparison of findings. For example, increasing field strength from 1.5 to 3 T increases the contrast to noise, “visibility rating,” and number of microbleeds detected by approximately 30% (60, 61). Recent work using 7 T MRI may further improve reliability of detection of MB (62), however one study with post-mortem correlation has suggested that at this field strength, non-hemorrhagic iron deposition may mimic microbleeds, resulting in poorer diagnostic specificity (63).

SWI increases the effect of conventional T2*GRE by image post-processing, multiplying magnetic resonance signal magnitude with the signal pulse shift. This provides greater contrast compared with T2* GRE, resulting in detection of 50–70% more lesions (59, 64, 65). However, whether this increase in lesion identification translates to a clinically meaningful difference is debated. Goos et al. found that although SWI enabled identification of nearly twice as many lesions as T2* GRE, this did not alter any of the clinical associations in multivariate analyses (64).

As differences in assessment of microbleeds can contribute to considerable heterogeneity in the literature, efforts have been made to standardize approaches to their reading and definition Inter-observer agreement for identification of microbleeds varies in the literature from *k* = 0.3–0.97 (66). To address this, rating scales divide lesions into certain or uncertain, as well as by location, with significant improvement in inter-rater reliability (66, 67). Greater reader confidence has also been reported when microbleeds are present on serial images, read sequentially (68). Microbleeds may also “disappear” over time – although it is unknown how often this is due to true physiological resorption, as opposed to an imaging artifact (69–72).

Attempts have also been made to improve detection of microbleeds using automated algorithms. Although these may be useful in screening for multiple lobar microbleeds, to date they have not replaced manual assessment as although sensitive, they tend to lack specificity (73, 74).

Microbleeds are frequently identified in association with other MRI evidence of cerebrovascular pathology, in particular markers of cerebral small vessel disease (SVD). Lobar and deep ICH, IS, particular LI, and white matter hyperintensities [WMH, seen with T2/Fluid Attenuated Inversion Recovery (FLAIR) MRI sequences], have each been demonstrated in association with microbleeds in diverse populations (69, 75, 76).

A relationship between microbleeds and severity of WMH, or leukoaraiosis, has been demonstrated in ischemic and hemorrhagic stroke patients (12, 69, 75–77), AD (13, 78, 79), VaD (80), and community-dwelling elderly (16, 23). Deep (16), diffuse (deep and lobar) (76), and strictly lobar MB (70) have each been associated with greater WMH. Just as the distribution of microbleeds appears to vary according to the etiology and severity of SVD, so too may the pattern of WMH. A posterior-predominant distribution of WMH has been reported in AD patients with microbleeds, and in individuals with lobar ICH, reflecting the predilection of CAA pathology in these areas at post-mortem (13, 81). These findings have not been universally reported, however, with some finding no difference in WMH distribution between CAA, AD, and NC individuals (82). This may be explained by the fact that WMH have been correlated with a spectrum of post-mortem findings from tissue rarefaction, to myelin and axonal loss and mild gliosis (83), and a diverse list of possible contributing pathologies, including neurodegeneration, inflammation, and hypoxia (84).

Microbleeds have also been associated with presence of hippocampal atrophy in a large study of cognitively normal individuals, an association possibly mediated by CAA in the presence of underlying AD-pathology (85). More recently, an association has also been reported between lobar microbleeds and prominent perivascular spaces in cerebral white matter, but not deep brain structures (86). They hypothesized that abluminal accumulation of β-amyloid may mediate dilatation of the perivascular space in patients with CAA.

### Amyloid imaging with positron emission tomography

Positron Emission Tomography imaging with *N*-methyl-[11C]2-(4-methylaminophenyl)-6-hydroxybenzothiazole, also known as Pittsburgh Compound B, or “PiB” was first used to demonstrate presence of fibrillar β-amyloid *in vivo* in individuals with AD (87). PiB can also highlight presence of β-amyloid in cerebral vessel walls, even in the absence of parenchymal plaques, both *in vitro* (88) and *in vivo* with subsequent post-mortem follow-up (89, 90). Patients with symptomatic ICH meeting criteria for probable CAA present with PiB retention midway between AD patients and controls (91, 92), with higher PiB retention in the occipital region (relative to other cortical regions) than AD patients, consistent with a previously reported predilection of CAA for posterior brain structures (43, 44).

**Figure 1:**
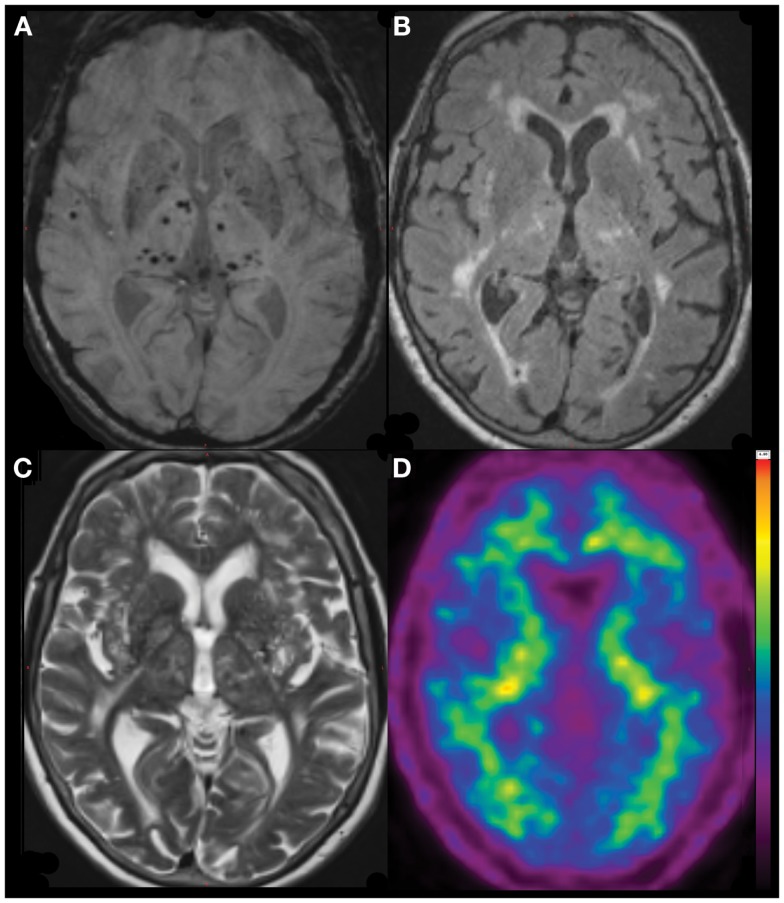
**Diffuse (predominantly non-lobar) Microbleeds in an 81-year-old lady referred with AD-type dementia (MMSE 22/30, CDR 1, CDR-SOB 5.5)**. SWI image **(A)**, with coregistered FLAIR **(B)**, T2 **(C)** and 11C-PiB PET **(D)** images demonstrating severe deep white matter hyperintensities but no significant beta-amyloid burden (neocortical SUVR = 1.2), suggesting that the presentation is due to severe cerebral small vessel disease, rather than Alzheimer’s disease.

**Figure 2:**
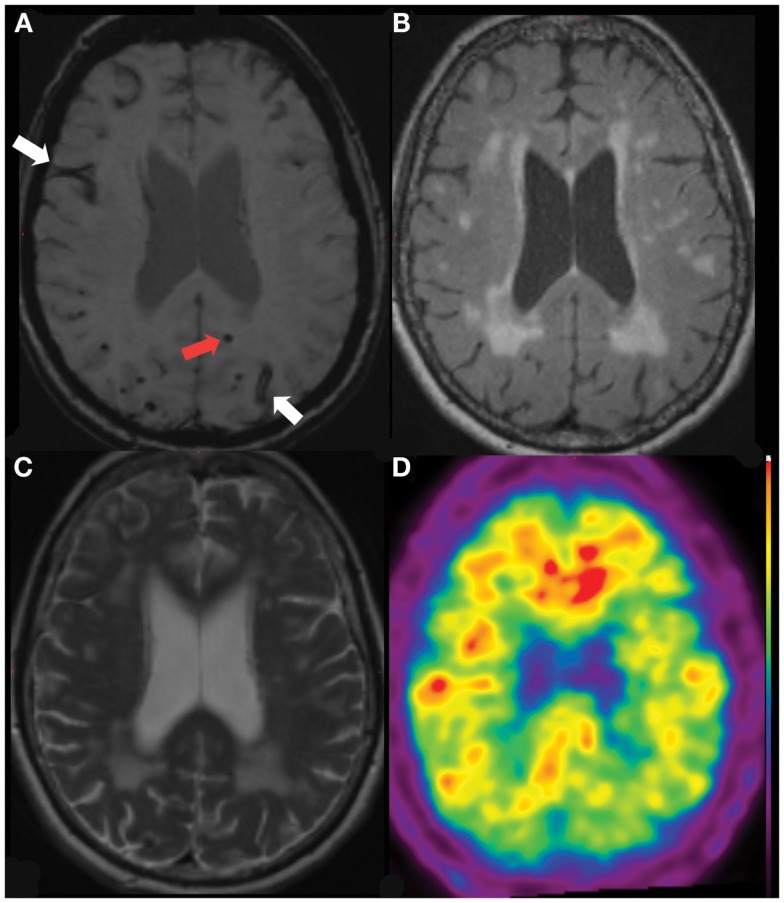
**Lobar Microbleed (red arrow) and Superficial Hemosiderosis (white arrows) in a 66-year-old lady referred initially with amnestic MCI (MMSE 22/30, CDR 0.5, CDR-SOB 4.5), subsequently diagnosed with AD-type dementia**. SWI image **(A)**, with coregistered FLAIR **(B)**, T2 **(C)** and 11C-PiB PET **(D)** images demonstrating severe deep white matter hyperintensities with elevated beta-amyloid burden (neocortical SUVR = 1.7).

In individuals with CAA, regions of increased PiB retention have been shown to coincide with sites of microbleeds, and with incident microbleeds at follow-up (93, 94). In cognitively normal controls, PiB retention has also been shown to correlate with lobar, but not deep microbleeds (36) (Figures 1 and 2). In multivariate analysis, PiB and age were independent predictors of lobar microbleeds, whereas presence of VRF, gender, and APOE were not. APOE ε4 carriage, a predictor of Lobar microbleeds in large population studies, is less strongly associated with LMB when adjusted for Aβ-burden, suggesting that the association between APOE ε4 and microbleeds may be mediated by Aβ (36, 95).

Use of molecular imaging for β-amyloid has shed new light on disease processes that were previously only identifiable at post mortem. However, currently available Aβ PET ligands are known to bind to fibrillar Aβ in both plaque and vessel walls. Hence, the relative contribution of each to the PET signal cannot be distinguished. A recent correlation observed between PiB and WMH volume in patients with probable CAA (but not AD or cognitively normal controls), might indicate that in some cases, much of the PiB signal may indeed be due to vascular, rather than parenchymal Aβ (96). Future work involving novel radioligands, selective for Aβ within vessel walls, have potential to clarify this (97, 98).

Use of other Aβ biomarkers, such as plasma and CSF assays have also been used to correlate with microbleeds. Profiles of Aβ40/42, total-tau, and phospho-tau may distinguish between AD, CAA, and NC (99) and between CAA and other causes of vascular disease (100).

## Clinical Features of Microbleeds

While microbleeds had previously been considered to be clinically silent, recent contributions to the literature have led to reassessment of their clinical and prognostic relevance.

### Prevalence and incidence

Table S1 in Supplementary Material outlines the prevalence of microbleeds in different clinical conditions, from community-dwelling elderly, free from cognitive impairment and neurologic disease, to ischemic and hemorrhagic cerebrovascular disease, different forms of dementia and genetic diseases.

In population studies, microbleeds are more prevalent with increasing age, from 6% aged 45–50 years, to 36% aged 80 or more (16). Individuals with no known vascular disease or risk factors may be at lower risk of microbleeds (prevalence 2.3%) (85).

In the setting of stroke, microbleeds are reported more frequently in ICH (prevalence 19–83%) than IS (15–35%) (10, 22, 29, 76, 77, 101–106). Of the IS subtypes, microbleeds occur more often in those with LI (LI, 26–62%) than atherothrombotic (21–46%) or cardio-embolic infarctions (4–30%) (22, 75, 76, 80, 104, 107). This is in keeping with post-mortem findings of small vessel lipohyalinosis in many individuals with microbleeds (33).

In comparison with cognitively normal controls (0–19%), microbleeds are more frequent in individuals with Mild Cognitive Impairment (MCI) (20–43%), patients with AD (18–32%), and VaD (65–85%) (13, 45, 72, 79, 80, 100, 108, 109). Microbleeds are also more prevalent individuals with progressive (31–54%), rather than stable MCI (36%) (72, 108), perhaps reflecting more severe SVD or AD-related pathology in these individuals.

Microbleeds have been reported in between 19 and 70% of individuals with CADASIL (19, 110, 111), and 67% of individuals with familial CAA (40). MB have also been reported in the setting of autoimmune encephalitides (112) and head trauma (65, 71, 113).

Few studies report rates of accumulation or incidence for microbleeds to date, with none that separate incident microbleeds by location. Lee prospectively reviewed 224 patients presenting with stroke or TIA over 3 years, reporting an incidence of 0.8 new microbleeds/year overall, increasing to 5.4/year in patients with multiple (≥5) baseline microbleeds (69). Another study of 26 patients with possible or probable CAA reported new microbleeds at 1 year in 46% of individuals (114), whereas the Rotterdam study of community-dwelling elderly reported an increase of prevalence of microbleeds from 24.4% at baseline to 28% at 3 years (70), although neither specify incidence *per se*.

It remains to be clarified whether microbleeds, once present, remain indefinitely, or if they regress over time. The majority of lesions appear to persist over serial scans, with only 1.4–2.3% of lesions “disappearing” (70, 115–117). However, others have reported lesions that appear to resolve progressively over time (69, 71). However, it is unclear whether microbleeds truly resolve, or if their “disappearance” is due to artifact or erroneous interpretation of baseline images (70). In one study of acute stroke patients, MB disappeared in a considerable 14.5% of cases, interestingly with disappearing lesions being associated with higher levels of LDL-cholesterol. The authors hypothesized that cholesterol levels may influence clearance of hemosiderin-laden macrophages, however also cautioned that these findings required validation in other studies (69).

In several longitudinal observational studies, the development of new microbleeds has been shown to be associated with presence of baseline microbleeds, markers of associated SVD (e.g., LI, ICH, WMH severity). These associations appear to hold true, despite diverse clinical populations studied (e.g., IS, ICH, or general population) (11, 69, 70, 114). In addition, in individuals with CAA, new microbleeds are associated with carriage of both APOE ε2 and ε4 (11).

When microbleeds are stratified by location, elevated systolic blood pressure (70, 118, 119), LI, and larger WMH volume predict incident deep or infratentorial microbleeds (70), whereas APOE ε4/ε4 genotype and larger WMH volume predict incident lobar microbleeds (70). Liu and colleagues noted that variability in blood pressure predicted microbleeds progression in deep and infratentorial regions only. They hypothesized that in deep and infratentorial regions, penetrating artery branches arise directly from large vessels and hence are more vulnerable to blood pressure variability than cortical vessels, where no association was seen (119).

### Microbleeds and alzheimer’s disease

Microbleeds are of significant interest in AD, both as an indication of risk of future hemorrhage, possible mediator of cognitive impairment, and more recently, as a marker of immunotherapy-related adverse events (Amyloid-Related Imaging Abnormalities, ARIA) (15).

In AD patients, while microbleeds have been associated with imaging markers of small vessel pathology such as WMH, they are less associated with strokes or VRF (78, 79). Microbleeds in AD are most often seen in cortico-subcortical distributions, and most individuals with AD have at least some CAA at post-mortem (44), which would implicate underlying CAA in the majority of microbleeds in AD.

Microbleeds are also recently described findings in treatment trials for AD, termed “Amyloid-Related Imaging Abnormalities” (“ARIA”). This term incorporates microHemorrhage and Hemosiderosis (ARIA-H) and vasogenic Edema and Effusions (ARIA-E), suggested to relate to altered Aβ trafficking in these individuals. ARIA gained attention following publication of phase I and II studies of bapineuzumab, a humanized monoclonal antibody specific to the N-terminal region of Aβ (14, 120, 121) although similar findings were also previously noted in human and animal reports with active immunotherapy and rarely, spontaneous inflammatory CAA (122–126).

Overall in the bapineuzumab study, ARIA-E were identified in 17% of cases, of whom 78% were asymptomatic. Coincident hemorrhage or hemosiderosis occurred in 47%. There was an increased risk of ARIA-E in patients treated with higher doses and in APOE ε4 homozygotes. There was no association between ARIA-E and age, gender, or WMH, and presence of microbleeds at baseline did not increase risk of incident ARIA-E (although participants with multiple microbleeds were excluded from participation). Although ARIA-E commonly preceded or coincided with ARIA-H, the two findings were not necessarily co-located, suggesting a generalized disruption of vascular integrity, rather than a focal insult. It is hypothesized that immunotherapy may precipitate failure of saturable perivascular Aβ clearance mechanisms by massive mobilization of soluble from sequestered Aβ. Hence, waste that is otherwise soluble (e.g., Aβ) accumulates, causing altered vascular permeability, and leakage of plasma and blood products (127).

ARIA-E has also been reported with other Aβ immunotherapeutic agents, albeit less frequently (128) and a gamma-secretase inhibitor (129), whereas by contrast, “spontaneous ARIA-E” is uncommon. Of 2762 baseline or screening MRI of mild-moderate AD patients enrolling in clinical trials, there were only four cases of VE, only one of which was associated with microbleeds (130).

Aside from being an incidental finding in AD patients, there is growing evidence that microbleeds may confer increased risk of cognitive impairment, and future cognitive decline. The effect of microbleeds may be due to focal damage or dysfunction, or be representative of more generalized processes, such as SVD from HT or CAA, or widespread β-amyloid pathology (24). Rosidi and colleagues proposed a model whereby microbleeds themselves, while not directly causing neuronal injury, leads to a sustained local inflammatory response, characterized by initial activation and persistent increase in microglia and macrophages, due to leakage of blood plasma into brain parenchyma. This inflammatory response appears to coincide with the extent of plasma leakage, supporting the hypothesis that this leakage may be the initiating event in the process, with ongoing inflammation then leading to neuronal dysfunction and cell death (131).

Studies into cognitive sequelae of microbleeds have been limited by heterogeneity in imaging parameters, sensitivity of cognitive measures, study demographics, and consequently, different patterns in distribution of microbleeds.

The majority of cross-sectional studies of the cognitive impact of microbleeds report finding impairments in executive function, with some also reporting decrements in attention, processing speed, and global cognition (132–135).

However, not all studies report cognitive associations according to location of microbleeds, which impedes comparison between cohorts. The Rotterdam Study reported independent associations between multiple (≥5) lobar microbleeds and all cognitive domains except memory. However deep microbleeds were not significantly associated with cognition, after adjusting for other possible confounding variables such as age and education (136). In the RUN-DMC study, deep microbleeds were associated with global cognition, psychomotor speed, and attention, whereas lobar microbleeds were associated with global cognition, memory, and executive function. Temporal lobe microbleeds were most associated with memory and attention, whereas frontal microbleeds were associated with memory, psychomotor speed, concept shifting, and attention (132). In a third study, Gregoire et al. reported that strictly lobar, but not deep microbleeds were associated with executive impairment in IS/TIA patients (137). In each study, the cognitive associations of microbleeds were independent of other markers of arteriosclerotic SVD, although there was no mechanism to adjust for presence of concomitant AD-pathology. It has, however, been shown with post-mortem histopathology that presence of moderate to severe CAA is linked with poorer cognitive scores (perceptual speed and episodic memory) during life even after correcting for presence of parenchymal Aβ-plaques (138). It could therefore be expected that some of the cognitive association of microbleeds may also be robust to adjustment for presence of parenchymal Aβ.

In AD patients, one study reported no difference in cognition between those with and without microbleeds (although there was a trend to poorer processing speed) (13), whereas in Subcortical VaD, microbleeds have been associated with impairment in numerous cognitive domains, including attention, verbal memory, visual memory, language, visuospatial function, and executive function. The large number of parietotemporal lesions in this cohort does suggest that some lesions may well be due to presence of CAA, and that parenchymal Aβ could be an important potential confounder driving some of these findings (45).

Microbleeds may also manifest in other cognitive or behavioral symptoms, according to their location. In 517 patients with IS, Tang (103) found an association between post-stroke emotional lability and microbleeds in the thalamus, but not other regions. No association was shown between emotional lability and WMH or infarcts.

There are few studies to date with illustrating the prognostic implication of microbleeds on cognition over time. Presence of microbleeds may predict the development of cognitive impairment in ICH patients (11), and conversely, in individuals with cognitive impairment post-stroke, those without microbleeds are four times more likely to revert to normal cognition than those with microbleeds (139).

Microbleeds have been predictive of cognitive deterioration in individuals with MCI in two studies (68, 72) but not another (108).

### Microbleeds, stroke, and vascular disease

Overall, microbleeds are associated with many risk factors for cerebrovascular pathology, including advancing age (23, 75, 140, 141), hypertension (10, 29, 75, 76, 111, 142), diabetes (111, 140), cigarette smoking (143). In one meta-analysis, hypertension, and diabetes remained significantly associated with microbleeds, whereas gender and smoking were not (144). Hypercholesterolemia is less strongly associated with microbleeds. In one study of ICH patients, low cholesterol level was associated with microbleeds (10), whereas other studies show no association (101, 142). In several studies, statin therapy for hypercholesterolemia has been implicated in increased risk of ICH (145, 146), although this has not been supported in a more recent meta-analysis (147).

Not all studies support an association between VRF and microbleeds. Both the large Framingham and AGES-Reykjavik cohorts failed to find any association (17, 141). The regional predominance of microbleeds in each study may have influenced these findings – for example, lobar microbleeds are not generally associated with VRF (16, 36) – although as these studies did not report lesion topography this can only be postulated.

Microbleeds are frequently identified in the setting of ICH, both in lobar or deep locations (11, 77, 148). Risk factors for microbleeds in ICH include advanced age, advanced leukoaraiosis, and lacunar infarcts (149). In individuals with ICH, a bimodal distribution of hemorrhage volume has been reported, with lesions tending to either <5 mm or >29 mm in diameter. Histopathologically, individuals with greatest microbleeds burden have increased vessel wall thickness compared with those with fewer microbleeds, however the prevalence of CAA-affected vessels does not differ (150).

Microbleeds are also common in individuals with IS, particularly with deep brain or lacunar infarcts, and atherosclerotic but not cardio-embolic disease (140). In IS patients, microbleeds are associated with advanced age, diabetes and prior use of antithrombotic drugs (140), and deep microbleeds have also been associated with hypertension (76). They are about four times more likely to be found in individuals with recurrent stroke than primary stroke, suggesting that they could be used as a prognostic marker (12, 107).

In longitudinal studies, microbleeds are predictive of future cerebrovascular events both in individuals with stroke, and in community-dwelling elderly (25, 104, 148, 151–153). Patients with IS or TIA with microbleeds are as much as three times more likely to have subsequent ICH (104, 140, 151, 152) including hemorrhagic transformation (154) or recurrent IS (152, 153). Other predictors of ICH in acute IS include age, NIHSS score, DM and lobar, cortico-subcortical distribution of microbleeds, with 2-year risk for ICH increasing from 0.5% if no baseline microbleeds, to 8% if ≥5 microbleeds (140, 155). ICH-related mortality also is associated with greater baseline numbers of microbleeds, and based on this study, the authors concluded that in individuals with multiple (≥5) microbleeds, the risk of ICH-related mortality (3.8%) may outweigh the potential benefit of antithrombotic therapy (Adjusted Risk Ratio 2.5–6%) (155). In individuals presenting with symptomatic ICH, presence of microbleeds are associated with increased risk of recurrent hemorrhage (10, 11), which may be at sites of prior MB, particularly in deep ICH (156). In community-dwelling elderly, microbleeds also are predictive of future stroke, particularly ICH. In one study with 3.6-year follow-up, 19% of subjects with baseline microbleeds had strokes, compared with 1% of those without (148).

Evidence from the PROGRESS trial suggests that blood pressure-lowering treatment in the setting of cerebrovascular disease is protective against both deep and lobar ICH. This suggests that as well as reducing risk of hypertensive ICH, antihypertensive therapy also reduces risk for ICH due to CAA (157). Although microbleeds were not analyzed, it could be inferred that individuals with microbleeds due to either process might similarly benefit.

Shortly after early studies reporting prevalence of microbleeds in patients with ICH and stroke, early data emerged to suggest that the natural history of these lesions may be influenced by use of antiplatelet, antithrombotic, and thrombolytic therapies. Several studies have identified increased frequency of microbleeds in antiplatelet medication users presenting with ICH (158–160), IS (80, 140, 161), and VaD (80). Subsequently a large population-based study also reported a higher prevalence of microbleeds in antiplatelet users, but not anticoagulant users (162).

In addition, prevalence of microbleeds has been noted to be higher in those with longer duration of antiplatelet use (161), and higher in those on aspirin than other agents (162, 163). However, other studies have not shown any association (36, 149).

Increasing evidence is emerging in studies with longitudinal follow-up, although overall the evidence is limited by number of studies and relative scarcity of incident hemorrhage. Huang et al. tracked IS patients for 18 months, all were treated with antiplatelet agents, either aspirin or cliostazol. Of 719 patients, 11 developed ICH, all of whom had prior microbleeds. In addition, aspirin users had higher ICH rates than those on cliostazol, suggesting that not all agents share the same risk (163). Biffi et al. followed 104 primary lobar ICH survivors prospectively for 15–57 months. Recurrent lobar ICH was associated with aspirin use, previous microbleeds, and posterior white matter hypodensity on CT (164). The most recent meta-analysis of antithrombotic and antiplatelet use with microbleeds included 1460 patients with ICH and 3817 with IS/TIA. Microbleeds were more common in ICH patients on either warfarin or antiplatelet agents, but not in IS/TIA patients. In 768 patients with longitudinal follow-up (90 ICH, 123 TIA, 555 IS), ICH were more common among users of (any) antithrombotic agents, but warfarin use specifically was not significant. The authors acknowledged several caveats, including significant heterogeneity between studies, and that adjustments had not been made for some potential confounders (e.g., presence of hypertension) (165).

The safety of newer antithrombotic agents (e.g., direct thrombin inhibitor, dabigatran, or factor Xa inhibitors rivaroxaban and apixaban) for non-valvular atrial fibrillation in individuals with microbleeds is at this stage unclear. To date, there is insufficient evidence to mandate withdrawal of any anticoagulant or antiplatelet agents, or use of alternative antithrombotic agents. Newer anticoagulants may confer reduced risk of ICH (166–168) however their lack of reversibility in setting of a putative bleeding event, and relative paucity of data in older patients is of some concern (169). Similarly, different antiplatelet agents may confer reduced ICH risk in IS patients with microbleeds [e.g., cliostazol versus aspirin, (163)], although more prospective data are urgently required to inform clinical decision-making (Table 2).

**Table 2:** **Key points**.

Microbleeds occur most commonly in the presence of cerebral small vessel disease, either arteriosclerosis or cerebral amyloid angiopathy
Although incidence rates for microbleeds have not been frequently described, microbleeds incidence relates to markers of severity of underlying disease, e.g., number of baseline microbleeds, severity of other SVD markers (e.g., lacunar infection and white matter hyperintensity)
Microbleeds are predictive of cognitive decline, intracerebral hemorrhage, ischemic deep brain infarction and death, however not all studies report location of microbleeds
As well as specifying MRI parameters, reporting of number and location of microbleeds is fundamental to interpretation of their clinical implications and enabling comparison across studies
The clinical outcome of microbleeds may be influenced by the use of antithrombotic medications, although prospective data are still limited. Current recommendations do not suggest withholding or changing therapy on basis of microbleeds, however future studies to address these questions are urgently required
Evidence suggests that adequate blood pressure control is important for reducing risk of ICH in individuals with microbleeds
Microbleeds in Alzheimer’s disease may signify presence of greater amounts of vascular Aβ deposition and loss of blood-brain barrier integrity. It is currently recommended that patients with four or more microbleeds are excluded from trials of Aβ immunotherapy

Several prospective studies [e.g., Clinical Relevance Of Microbleeds In Stroke study, CROMIS-2, (170)] have been proposed to address this evidence gap, aiming to recruit large cohorts with non-valvular atrial fibrillation and ICH, comparing incidence of cerebrovascular events according to presence of microbleeds and anticoagulant therapies. Results from these and similar studies are awaited with great interest.

In patients treated with thrombolysis for stroke or myocardial infarction, ICH is a feared complication, occurring in as many as 5.9% of cases (171). Concerns about safety of thrombolysis in individuals with microbleeds were raised with early series (172, 173). Subsequently, the large BRASIL (Bleeding Risk Analysis in Stroke Imaging before thromboLysis) study which analyzed MRI data from 570 IS patients in 13 centers in Europe, North America, and Asia, failed to find a significant association between baseline microbleeds and thrombolysis-associated ICH (102). However, there has been some criticism leveled at the study’s conclusions, including that it was underpowered, and did not stratify microbleeds by distribution. The prevalence of microbleeds was only 15%, somewhat lower than the majority of other IS studies, and there were few patients (only six) with multiple microbleeds.

In two recent meta-analyses, presence of microbleeds was associated with a trend to increased risk of ICH post-thrombolysis, and there was a significant relationship between microbleeds burden and symptomatic ICH. However to date, interpretation of the literature is limited by heterogeneity in study size and design, and overall, each conclude that there is currently insufficient evidence to exclude patients with microbleeds from thrombolysis (174, 175).

A relationship between microbleeds and other manifestations of hypertensive vascular damage has also been reported, including peripheral arterial stiffness (176) (in a sample with a greater proportion of deep microbleeds), and left ventricular hypertrophy with deep (but not lobar) microbleeds (177). Impaired cerebrovascular reactivity with 7 T functional MRI has also been reported with microbleeds in patients with atherosclerotic disease. Interestingly, the majority of microbleeds in this sample were in a lobar distribution, implicating CAA, rather than deep arteriosclerosis, as the underlying mechanism behind this reduction in vasoactivity (62).

### Microbleeds and motor changes

Deep brain microbleeds have also been linked with gait disturbance, such as reduced stride length, and impaired timed-up-and-go test. In particular, microbleeds proximal to major motor pathways, such as in the basal ganglia, thalamus, and frontal lobes, showed the strongest association with gait change. However, a link between temporal lobe microbleeds and reduced gait speed is also reported, a finding not directly explicable by anatomical pathways, which suggests that more widespread neuronal disruption may be present in these subjects (178). Individuals with multiple microbleeds due to CAA may present with cortical motor symptoms such as hemiparesis, dysphasia, or seizures (179–181), with associated findings of white matter change on MRI suggestive of vasogenic edema. These symptoms may be responsive to corticosteroid therapy (123).

### Microbleeds and mortality risk

Prospective data on the microbleeds and mortality are limited. In 2004, Greenberg et al. identified and association between baseline hemorrhage burden (including microbleeds) and a composite endpoint, including death, cognitive impairment, or loss of independent functioning (11) in patients with ICH. In the PROspective Study of Pravastatin in the Elderly at Risk (PROSPER), 435 individuals with VRF or vascular disease were followed for 7 years. Over the study period, microbleeds were associated with a sixfold increased risk of stroke-related death. Individuals with non-lobar microbleeds had double the risk of cardiovascular disease-related death (but not stroke-related death), independent of VRF, whereas those with lobar microbleeds had a sevenfold increase in the risk of stroke-related death, but not cardiovascular death (142).

## Conclusion

Only relatively recently identified, microbleeds are increasingly appreciated as a marker of underlying disease states and risk for ischemic and hemorrhagic sequelae, and cognitive decline. However several questions remain to be clarified, including the rate of incidence for these lesions in aging and different disease states, and to what extent different therapies modify this rate. Further, it is still not clear whether microbleeds themselves are responsible for altered cognition in AD and cerebrovascular disease, or if they are simply a marker for the underlying pathology, namely hypertensive SVD (in cerebrovascular disease), or fibrillar β-amyloid deposition (in AD). Our understanding of their prognostic implications will continue to improve with ongoing longitudinal assessment.

## Conflict of Interest Statement

The authors declare that the research was conducted in the absence of any commercial or financial relationships that could be construed as a potential conflict of interest.

## Supplementary Material

The Supplementary Material for this article can be found online at: http://www.frontiersin.org/journal/10.3389/fneur.2013.00205/abstract

## References

[1] Greenberg SM Cerebral amyloid angiopathy: prospects for clinical diagnosis and treatment. Neurology (1998) 51:690–4 10.1212/WNL.51.3.690 9748011

[2] Flaherty ML Haverbusch M Sekar P Kissela B Kleindorfer D Moomaw CJ Long-term mortality after intracerebral hemorrhage. Neurology (2006) 66:1182–6 10.1212/01.wnl.0000208400.08722.7c 16636234

[3] Qureshi AI Suri MFK Mohammad Y Guterman LR Hopkins LN Isolated and borderline isolated systolic hypertension relative to long-term risk and type of stroke: a 20-year follow-up of the national health and nutrition survey. Stroke (2002) 33:2781–8 10.1161/01.STR.0000039402.05613.0F 12468770

[4] Kurth T Kase CS Berger K Gaziano JM Cook NR Buring JE Smoking and risk of hemorrhagic stroke in women. Stroke (2003) 34:2792–5 10.1161/01.STR.0000065200.93070.32 14615625

[5] Matsukawa H Shinoda M Fujii M Takahashi O Yamamoto D Murakata A Factors associated with lobar vs. non-lobar intracerebral hemorrhage. Acta Neurol Scand (2011) [cited 2012 Feb 29]. Available from: http://www.ncbi.nlm.nih.gov/pubmed/22067041 10.1111/j.1600-0404.2011.01615.x 22067041

[6] Hillbom M Saloheimo P Juvela S Alcohol consumption, blood pressure, and the risk of stroke. Curr Hypertens Rep (2011) 13:208–13 10.1007/s11906-011-0194-y 21327566

[7] Ariesen MJ Claus SP Rinkel GJE Algra A Risk factors for intracerebral hemorrhage in the general population: a systematic review. Stroke (2003) 34:2060–5 10.1161/01.STR.0000080678.09344.8D 12843354

[8] Pantoni L Cerebral small vessel disease: from pathogenesis and clinical characteristics to therapeutic challenges. Lancet Neurol (2010) 9:689–701 10.1016/S1474-4422(10)70104-6 20610345

[9] Foulkes MA Wolf PA Price TR Mohr JP Hier DB The Stroke Data Bank: design, methods, and baseline characteristics. Stroke (1988) 19:547–54 10.1161/01.STR.19.5.547 3363586

[10] Jeon S-B Kang D-W Cho A-H Lee E-M Choi CG Kwon SU Initial microbleeds at MR imaging can predict recurrent intracerebral hemorrhage. J Neurol (2007) 254:508–12 10.1007/s00415-006-0406-6 17401517

[11] Greenberg SM Eng JA Ning M Smith EE Rosand J Hemorrhage burden predicts recurrent intracerebral hemorrhage after lobar hemorrhage. Stroke (2004) 35:1415–20 10.1161/01.STR.0000126807.69758.0e 15073385

[12] Gao T Wang Y Zhang Z Silent cerebral microbleeds on susceptibility-weighted imaging of patients with ischemic stroke and leukoaraiosis. Neurol Res (2008) 30:272–6 10.1179/016164107X251556 18384712

[13] Pettersen JA Sathiyamoorthy G Gao F-Q Szilagyi G Nadkarni NK St George-Hyslop P Microbleed topography, leukoaraiosis, and cognition in probable Alzheimer disease from the Sunnybrook dementia study. Arch Neurol (2008) 65:790–5 10.1001/archneur.65.6.790 18541799

[14] Rinne JO Brooks DJ Rossor MN Fox NC Bullock R Klunk WE 11C-PiB PET assessment of change in fibrillar amyloid-beta load in patients with Alzheimer’s disease treated with bapineuzumab: a phase 2, double-blind, placebo-controlled, ascending-dose study. Lancet Neurol (2010) 9:363–72 10.1016/S1474-4422(10)70043-0 20189881

[15] Sperling RA Jack CR Jr Black SE Frosch MP Greenberg SM Hyman BT Amyloid-related imaging abnormalities in amyloid-modifying therapeutic trials: recommendations from the Alzheimer’s Association Research Roundtable Workgroup. Alzheimers Dement (2011) 7:367–85 10.1016/j.jalz.2011.05.2351 21784348 PMC3693547

[16] Poels MMF Vernooij MW Ikram MA Hofman A Krestin GP van der Lugt A Prevalence and risk factors of cerebral microbleeds: an update of the Rotterdam scan study. Stroke (2010) 41:S103–6 10.1161/STROKEAHA.110.595181 20876479

[17] Sveinbjornsdottir S Sigurdsson S Aspelund T Kjartansson O Eiriksdottir G Valtysdottir B Cerebral microbleeds in the population based AGES-Reykjavik study: prevalence and location. J Neurol Neurosurg Psychiatry (2008) 79:1002–6 10.1136/jnnp.2007.121913 18270235 PMC11090473

[18] Blitstein MK Tung GA MRI of cerebral microhemorrhages. AJR Am J Roentgenol (2007) 189:720–5 10.2214/AJR.07.2249 17715122

[19] Dichgans M Holtmannspötter M Herzog J Peters N Bergmann M Yousry TA Cerebral microbleeds in CADASIL. Stroke (2002) 33:67–71 10.1161/hs0102.100885 11779891

[20] Roob G Fazekas F Magnetic resonance imaging of cerebral microbleeds. Curr Opin Neurol (2000) 13:69–73 10.1097/00019052-200002000-00013 10719653

[21] Kwa VI Franke CL Verbeeten B Jr Stam J Silent intracerebral microhemorrhages in patients with ischemic stroke. Amsterdam Vascular Medicine Group. Ann Neurol (1998) 44:372–7 9749604 10.1002/ana.410440313

[22] Kato H Izumiyama M Izumiyama K Takahashi A Itoyama Y Silent cerebral microbleeds on T2*-weighted MRI: correlation with stroke subtype, stroke recurrence, and leukoaraiosis. Stroke (2002) 33:1536–40 10.1161/01.STR.0000018012.65108.86 12052987

[23] Takashima Y Mori T Hashimoto M Kinukawa N Uchino A Yuzuriha T Clinical correlating factors and cognitive function in community-dwelling healthy subjects with cerebral microbleeds. J Stroke Cerebrovasc Dis (2011) 20:105–10 10.1016/j.jstrokecerebrovasdis.2009.11.007 20580259

[24] Werring DJ Gregoire SM Cipolotti L Cerebral microbleeds and vascular cognitive impairment. J Neurol Sci (2010) 299:131–5 10.1016/j.jns.2010.08.034 20850134

[25] Nishikawa T Ueba T Kajiwara M Fujisawa I Miyamatsu N Yamashita K Cerebral microbleeds predict first-ever symptomatic cerebrovascular events. Clin Neurol Neurosurg (2009) 111:825–8 10.1016/j.clineuro.2009.08.011 19765890

[26] Senior K Microbleeds may predict cerebral bleeding after stroke. Lancet (2002) 359:769 10.1016/S0140-6736(02)07911-4 11888596

[27] Atlas SW Mark AS Grossman RI Gomori JM Intracranial hemorrhage: gradient-echo MR imaging at 1.5 T. Comparison with spin-echo imaging and clinical applications. Radiology (1988) 168:803–7 3406410 10.1148/radiology.168.3.3406410

[28] Wu Z Mittal S Kish K Yu Y Hu J Haacke EM Identification of calcification with magnetic resonance imaging using susceptibility-weighted imaging: a case study. J Magn Reson Imaging (2009) 29:177–82 10.1002/jmri.21617 19097156 PMC2646180

[29] Tanaka A Ueno Y Nakayama Y Takano K Takebayashi S Small chronic hemorrhages and ischemic lesions in association with spontaneous intracerebral hematomas. Stroke (1999) 30:1637–42 10.1161/01.STR.30.8.1637 10436114

[30] Vernooij MW Ikram MA Hofman A Krestin GP Breteler MMB van der Lugt A Superficial siderosis in the general population. Neurology (2009) 73:202–5 10.1212/WNL.0b013e3181ae7c5e 19620607

[31] De Reuck J Deramecourt V Cordonnier C Leys D Pasquier F Maurage C-A Prevalence of small cerebral bleeds in patients with a neurodegenerative dementia: a neuropathological study. J Neurol Sci (2011) 300:63–6 10.1016/j.jns.2010.09.031 20965516

[32] Fazekas F Kleinert R Roob G Kleinert G Kapeller P Schmidt R Histopathologic analysis of foci of signal loss on gradient-echo T2*-weighted MR images in patients with spontaneous intracerebral hemorrhage: evidence of microangiopathy-related microbleeds. Am J Neuroradiol (1999) 20:637–42 10319975 PMC7056037

[33] Shoamanesh A Kwok CS Benavente O Cerebral microbleeds: histopathological correlation of neuroimaging. Cerebrovasc Dis (2011) 32:528–34 10.1159/000331466 22104448

[34] Vernooij MW van der Lugt A Ikram MA Wielopolski PA Niessen WJ Hofman A Prevalence and risk factors of cerebral microbleeds: the Rotterdam Scan Study. Neurology (2008) 70:1208–14 10.1212/01.wnl.0000307750.41970.d9 18378884

[35] Kim M Bae HJ Lee J Kang L Lee S Kim S APOE epsilon2/epsilon4 polymorphism and cerebral microbleeds on gradient-echo MRI. Neurology (2005) 65:1474–5 10.1212/01.wnl.0000183311.48144.7f 16275840

[36] Yates PA Sirisriro R Villemagne VL Farquharson S Masters CL Rowe CC Cerebral microhemorrhage and brain β-amyloid in aging and Alzheimer disease. Neurology (2011) 77:48–54 10.1212/WNL.0b013e318221ad36 21700585

[37] Imaizumi T Miyata K Inamura S Kohama I Nyon KS Nomura T The difference in location between traumatic cerebral microbleeds and microangiopathic microbleeds associated with stroke. J Neuroimaging (2011) 21:359–64 10.1111/j.1552-6569.2011.00593.x 21447027

[38] Itoh Y Yamada M Hayakawa M Otomo E Miyatake T Cerebral amyloid angiopathy: a significant cause of cerebellar as well as lobar cerebral hemorrhage in the elderly. J Neurol Sci (1993) 116:135–41 10.1016/0022-510X(93)90317-R 8336159

[39] Knudsen KA Rosand J Karluk D Greenberg SM Clinical diagnosis of cerebral amyloid angiopathy: validation of the Boston criteria. Neurology (2001) 56:537–539 10.1212/WNL.56.4.537 11222803

[40] van Rooden S van der Grond J van den Boom R Haan J Linn J Greenberg SM Descriptive analysis of the Boston criteria applied to a Dutch-type cerebral amyloid angiopathy population. Stroke (2009) 40:3022–7 10.1161/STROKEAHA.109.554378 19556530

[41] Linn J Halpin A Demaerel P Ruhland J Giese AD Dichgans M Prevalence of superficial siderosis in patients with cerebral amyloid angiopathy. Neurology (2010) 74:1346–50 10.1212/WNL.0b013e3181dad605 20421578 PMC2875936

[42] Von sattel JP Myers RH Hedley-Whyte ET Ropper AH Bird ED Richardson EP Jr Cerebral amyloid angiopathy without and with cerebral hemorrhages: a comparative histological study. Ann Neurol (1991) 30:637–649 10.1002/ana.410300503 1763890

[43] Rosand J Muzikansky A Kumar A Wisco JJ Smith EE Betensky RA Spatial clustering of hemorrhages in probable cerebral amyloid angiopathy. Ann Neurol (2005) 58:459–62 10.1002/ana.20596 16130107

[44] Vinters H Gilbert J Cerebral amyloid angiopathy: incidence and complications in the aging brain. II. The distribution of amyloid vascular changes. Stroke (1983) 14:924–8 10.1161/01.STR.14.6.924 6658996

[45] Won Seo S Hwa Lee B Kim E-J Chin J Sun Cho Y Yoon U Clinical significance of microbleeds in subcortical vascular dementia. Stroke (2007) 38:1949–51 10.1161/STROKEAHA.106.477315 17510457

[46] Chalmers K Wilcock GK Love S APOEe4 influences the pathological phenotype of Alzheimer’s disease by favouring cerebrovascular over parenchymal accumulation of Abeta protein. Neuropathol Appl Neurobiol (2003) 29:231–8 10.1046/j.1365-2990.2003.00457.x 12787320

[47] Sullivan PM Mace BE Estrada JC Schmechel DE Alberts MJ Human apolipoprotein E4 targeted replacement mice show increased prevalence of intracerebral hemorrhage associated with vascular amyloid deposition. J Stroke Cerebrovasc Dis (2008) 17:303–11 10.1016/j.jstrokecerebrovasdis.2008.03.011 18755411

[48] Trembath D Ervin JF Broom L Szymanski M Welsh-Bohmer K Pieper C The distribution of cerebrovascular amyloid in Alzheimer’s disease varies with ApoE genotype. Acta Neuropathol (2006) 113:23–31 10.1007/s00401-006-0162-9 17089130

[49] McCarron MO Nicoll JA Stewart J Ironside JW Mann DM Love S The apolipoprotein E epsilon2 allele and the pathological features in cerebral amyloid angiopathy-related hemorrhage. J Neuropathol Exp Neurol (1999) 58:711–8 10.1097/00005072-199907000-00005 10411341

[50] Maxwell SS Jackson CA Paternoster L Cordonnier C Thijs V Al-Shahi Salman R Genetic associations with brain microbleeds: systematic review and meta-analyses. Neurology (2011) 77:158–67 10.1212/WNL.0b013e318224afa3 21715706 PMC3140069

[51] Yamada M Cerebral amyloid angiopathy and gene polymorphisms. J Neurol Sci (2004) 226:41–4 10.1016/j.jns.2004.09.009 15537517

[52] Biffi A Shulman JM Jagiella JM Cortellini L Ayres AM Schwab K Genetic variation at CR1 increases risk of cerebral amyloid angiopathy. Neurology (2012) 78:334–41 10.1212/WNL.0b013e3182452b40 22262751 PMC3280047

[53] Joutel A Bousser M-G Biousse V Labauge P Chabriat H Nibbio A A gene for familial hemiplegic migraine maps to chromosome 19. Nat Genet (1993) 5:40–5 10.1038/ng0993-40 8220421

[54] Levy E Carman MD Fernandez-Madrid IJ Power MD Lieberburg I van Duinen SG Mutation of the Alzheimer’s disease amyloid gene in hereditary cerebral hemorrhage, Dutch type. Science (1990) 248:1124–6 10.1126/science.2111584 2111584

[55] Grabowski TJ Cho HS Vonsattel JP Rebeck GW Greenberg SM Novel amyloid precursor protein mutation in an Iowa family with dementia and severe cerebral amyloid angiopathy. Ann Neurol (2001) 49:697–705 10.1002/ana.1009 11409420

[56] Ryan NS Bastos-Leite AJ Rohrer JD Werring DJ Fox NC Rossor MN Cerebral microbleeds in familial Alzheimer’s disease. Brain (2012) 135(1):e201 10.1093/brain/awr126 21685457 PMC3859452

[57] Saint-Aubert L Planton M Hannequin D Albucher J-F Delisle M-B Payoux P Amyloid imaging with AV45 (^18^F-florbetapir) in a cognitively normal AβPP duplication carrier. J Alzheimers Dis (2012) 28:877–83 10.3233/JAD-2011-111598 22156048

[58] Liu T Surapaneni K Lou M Cheng L Spincemaille P Wang Y Cerebral microbleeds: burden assessment by using quantitative susceptibility mapping. Radiology (2012) 262:269–78 10.1148/radiol.11110251 22056688 PMC3244668

[59] Nandigam RNK Viswanathan A Delgado P Skehan ME Smith EE Rosand J MR imaging detection of cerebral microbleeds: effect of susceptibility-weighted imaging, section thickness, and field strength. AJNR Am J Neuroradiol (2009) 30:338–43 10.3174/ajnr.A1355 19001544 PMC2760298

[60] Stehling C Wersching H Kloska SP Kirchhof P Ring J Nassenstein I Detection of asymptomatic cerebral microbleeds: a comparative study at 1.5 and 3.0 T. Acad Radiol (2008) 15:895–900 10.1016/j.acra.2008.01.013 18572126

[61] Tatsumi S Ayaki T Shinohara M Yamamoto T Type of gradient recalled-echo sequence results in size and number change of cerebral microbleeds. AJNR Am J Neuroradiol (2008) 29:e13–13 10.3174/ajnr.A0908 18184838 PMC7978196

[62] Conijn MMA Hoogduin JM van der Graaf Y Hendrikse J Luijten PR Geerlings MI Microbleeds, lacunar infarcts, white matter lesions and cerebrovascular reactivity – a 7 T study. Neuroimage (2012) 59:950–6 10.1016/j.neuroimage.2011.08.059 21930217

[63] De Reuck J Caparros-Lefebvre D Deramecourt V Maurage CA Hippocampal microbleed on a post-mortem t(2)*-weighted gradient-echo 7.0-tesla magnetic resonance imaging? Case Rep Neurol (2011) 3:223–6 10.1159/000332611 22121349 PMC3223029

[64] Goos JDC van der Flier WM Knol DL Pouwels PJW Scheltens P Barkhof F Clinical relevance of improved microbleed detection by susceptibility-weighted magnetic resonance imaging. Stroke (2011) 42:1894–900 10.1161/STROKEAHA.110.599837 21566235

[65] Akiyama Y Miyata K Harada K Minamida Y Nonaka T Koyanagi I Susceptibility-weighted magnetic resonance imaging for the detection of cerebral microhemorrhage in patients with traumatic brain injury. Neurol Med Chir (Tokyo) (2009) 49:97–9 discussion 99, 10.2176/nmc.49.97 19318732

[66] Cordonnier C Potter GM Jackson CA Doubal F Keir S Sudlow CLM Improving interrater agreement about brain microbleeds. Stroke (2009) 40:94–9 10.1161/STROKEAHA.108.526996 19008468

[67] Gregoire SM Chaudhary UJ Brown MM Yousry TA Kallis C Jäger HR The Microbleed Anatomical Rating Scale (MARS): reliability of a tool to map brain microbleeds. Neurology (2009) 73:1759–66 10.1212/WNL.0b013e3181c34a7d 19933977

[68] Ayaz M Boikov AS Haacke EM Kido DK Kirsch WM Imaging cerebral microbleeds using susceptibility weighted imaging: one step toward detecting vascular dementia. J Magn Reson Imaging (2010) 31:142–8 10.1002/jmri.22001 20027582 PMC2802499

[69] Lee S-H Lee S-T Kim BJ Park H-K Kim C-K Jung K-H Dynamic temporal change of cerebral microbleeds: long-term follow-up MRI study. PLoS One (2011) 6:e25930 10.1371/journal.pone.0025930 22022473 PMC3191164

[70] Poels MMF Ikram MA van der Lugt A Hofman A Krestin GP Breteler MMB Incidence of cerebral microbleeds in the general population: the Rotterdam Scan Study. Stroke (2011) 42:656–61 10.1161/STROKEAHA.110.607184 21307170

[71] Asif IM Harmon KG Drezner JA O’Kane JW Cerebral microhemorrhages in a collegiate football player. Sports Health (2010) 2(5):391–4 10.1177/1941738110374628 23015965 PMC3445056

[72] Kirsch W McAuley G Holshouser B Petersen F Ayaz M Vinters HV Serial susceptibility weighted MRI measures brain iron and microbleeds in dementia. J Alzheimers Dis (2009) 17:599–609 10.3233/JAD-2009-1073 19433895 PMC2788087

[73] Fazlollahi A Meriaudau F Giancardo L Desmond PM Villemagne VL Rowe CC Automatic detection of cerebral microbleed in SWI using radon transform. Proceedings of ISMRM 2013 (2013).

[74] Seghier ML Kolanko MA Leff AP Jäger HR Gregoire SM Werring DJ Microbleed detection using automated segmentation (MIDAS): a new method applicable to standard clinical MR images. PLoS One (2011) 6:e17547 10.1371/journal.pone.0017547 21448456 PMC3063172

[75] Tsushima Y Aoki J Endo K Brain microhemorrhages detected on T2*-weighted gradient-echo MR images. AJNR Am J Neuroradiol (2003) 24:88–96 12533332 PMC8148967

[76] Yakushiji Y Yokota C Yamada N Kuroda Y Minematsu K Clinical characteristics by topographical distribution of brain microbleeds, with a particular emphasis on diffuse microbleeds. J Stroke Cerebrovasc Dis (2010) [cited 2011 Feb 1]. Available from: http://www.ncbi.nlm.nih.gov/pubmed/20621512 10.1016/j.jstrokecerebrovasdis.2009.12.001 20621512

[77] Alemany M Stenborg A Terent A Sonninen P Raininko R Coexistence of microhemorrhages and acute spontaneous brain hemorrhage: correlation with signs of microangiopathy and clinical data. Radiology (2006) 238:240–7 10.1148/radiol.2381040551 16373772

[78] Nakata-Kudo Y Mizuno T Yamada K Shiga K Yoshikawa K Mori S Microbleeds in Alzheimer disease are more related to cerebral amyloid angiopathy than cerebrovascular disease. Dement Geriatr Cogn Disord (2006) 22:8–14 10.1159/000092958 16645275

[79] Hanyu H Tanaka Y Shimizu S Takasaki M Abe K Cerebral microbleeds in Alzheimer’s disease. J Neurol (2003) 250:1496–7 10.1007/s00415-003-0245-7 14673587

[80] Hanyu H Tanaka Y Shimizu S Takasaki M Fujita H Kaneko N Cerebral microbleeds in Binswanger’s disease: a gradient-echo T2*-weighted magnetic resonance imaging study. Neurosci Lett (2003) 340:213–6 10.1016/S0304-3940(03)00121-6 12672544

[81] Zhu Y-C Chabriat H Godin O Dufouil C Rosand J Greenberg SM Distribution of white matter hyperintensity in cerebral hemorrhage and healthy aging. J Neurol (2011) [cited 2011 Dec 5]. Available from: http://www.ncbi.nlm.nih.gov/pubmed/21877206 10.1007/s00415-011-6218-3 PMC3658108 21877206

[82] Holland CM Smith EE Csapo I Gurol ME Brylka DA Killiany RJ Spatial distribution of white-matter hyperintensities in Alzheimer disease. Stroke (2008) 39:1127–33 10.1161/STROKEAHA.107.497438 18292383 PMC2754400

[83] Pantoni L Garcia JH Pathogenesis of leukoaraiosis: a review. Stroke (1997) 28:652–9 10.1161/01.STR.28.3.652 9056627

[84] Gouw A Seewann A van der Flier W Barkhof F Rozemuller A Scheltens P Heterogeneity of small vessel disease: a systematic review of MRI and histopathology correlations. J Neurol Neurosurg Psychiatry (2011) 82:126–35 10.1136/jnnp.2009.204685 20935330

[85] Chowdhury MH Nagai A Bokura H Nakamura E Kobayashi S Yamaguchi S Age-related changes in white matter lesions, hippocampal atrophy, and cerebral microbleeds in healthy subjects without major cerebrovascular risk factors. J Stroke Cerebrovasc Dis (2011) 20:302–9 10.1016/j.jstrokecerebrovasdis.2009.12.010 20634092

[86] Martinez-Ramirez S Pontes-Neto OM Dumas AP Auriel E Halpin A Quimby M Topography of dilated perivascular spaces in subjects from a memory clinic cohort. Neurology (2013) 80:1551–6 10.1212/WNL.0b013e31828f1876 23553482 PMC3662325

[87] Klunk WE Engler H Nordberg A Wang Y Blomqvist G Holt DP Imaging brain amyloid in Alzheimer’s disease with Pittsburgh Compound-B. Ann Neurol (2004) 55:306–19 10.1002/ana.20009 14991808

[88] Lockhart A Lamb JR Osredkar T Sue LI Joyce JN Ye L PIB is a non-specific imaging marker of amyloid-beta (Abeta) peptide-related cerebral amyloidosis. Brain (2007) 130:2607–15 10.1093/brain/awm191 17698496

[89] Ikonomovic MD Klunk WE Abrahamson EE Mathis CA Price JC Tsopelas ND Post-mortem correlates of in vivo PiB-PET amyloid imaging in a typical case of Alzheimer’s disease. Brain (2008) 131:1630–45 10.1093/brain/awn016 18339640 PMC2408940

[90] Greenberg SM Grabowski T Gurol ME Skehan ME Nandigam RNK Becker JA Detection of isolated cerebrovascular beta-amyloid with Pittsburgh compound B. Ann Neurol (2008) 64:587–91 10.1002/ana.21528 19067370 PMC2605158

[91] Johnson KA Gregas M Becker JA Kinnecom C Salat DH Moran EK Imaging of amyloid burden and distribution in cerebral amyloid angiopathy. Ann Neurol (2007) 62:229–34 10.1002/ana.21164 17683091

[92] Ly JV Donnan GA Villemagne VL Zavala JA Ma H O’Keefe G 11C-PIB binding is increased in patients with cerebral amyloid angiopathy-related hemorrhage. Neurology (2010) 74:487–93 10.1212/WNL.0b013e3181cef7e3 20142615

[93] Dierksen GA Skehan ME Khan MA Jeng J Nandigam RNK Becker JA Spatial relation between microbleeds and amyloid deposits in amyloid angiopathy. Ann Neurol (2010) 68:545–8 10.1002/ana.22099 20865701 PMC2964411

[94] Gurol ME Dierksen G Betensky R Gidicsin C Halpin A Becker A Predicting sites of new hemorrhage with amyloid imaging in cerebral amyloid angiopathy. Neurology (2012) 79:320–6 10.1212/WNL.0b013e31826043a9 22786597 PMC3400097

[95] Kantarci K Gunter JL Tosakulwong N Weigand SD Senjem MS Petersen RC Focal hemosiderin deposits and β-amyloid load in the ADNI cohort. Alzheimers Dement (2013) [cited 2013 Feb 28]. Available from: http://www.sciencedirect.com/science/article/pii/S1552526012025733 10.1016/j.jalz.2012.10.011 PMC3770782 23375568

[96] Gurol ME Viswanathan A Gidicsin C Hedden T Martinez-Ramirez S Dumas A Cerebral amyloid angiopathy burden associated with leukoaraiosis: a positron emission tomography/magnetic resonance imaging study. Ann Neurol (2012). 10.1002/ana.23830 23424091 PMC3715595

[97] Han BH Zhou M Vellimana AK Milner E Kim DH Greenberg JK Resorufin analogs preferentially bind cerebrovascular amyloid: potential use as imaging ligands for cerebral amyloid angiopathy. Mol Neurodegener (2011) 6:86 10.1186/1750-1326-6-86 22192811 PMC3259047

[98] Zha Z Choi SR Ploessl K Lieberman BP Qu W Hefti F Multidentate (18)F-polypegylated styrylpyridines as imaging agents for Aβ plaques in cerebral amyloid angiopathy (CAA). J Med Chem (2011) 54:8085–98 10.1021/jm2009106 22011144 PMC3228909

[99] Verbeek MM Kremer BPH Rikkert MO Van Domburg PHMF Skehan ME Greenberg SM Cerebrospinal fluid amyloid beta(40) is decreased in cerebral amyloid angiopathy. Ann Neurol (2009) 66:245–9 10.1002/ana.21694 19743453 PMC3697750

[100] Goos JDC Teunissen CE Veerhuis R Verwey NA Barkhof F Blankenstein MA Microbleeds relate to altered amyloid-beta metabolism in Alzheimer’s disease. Neurobiol Aging (2012) 33:.e1–1011 10.1016/j.neurobiolaging.2011.10.026 22118945

[101] Orken DN Kenangil G Uysal E Gundogdu L Erginoz E Forta H Lack of association between cerebral microbleeds and low serum cholesterol in patients with acute intracerebral hemorrhage. Clin Neurol Neurosurg (2010) 112:668–71 10.1016/j.clineuro.2010.05.004 20627554

[102] Fiehler J Albers GW Boulanger J-M Derex L Gass A Hjort N Bleeding risk analysis in stroke imaging before thromboLysis (BRASIL): pooled analysis of T2*-weighted magnetic resonance imaging data from 570 patients. Stroke (2007) 38:2738–44 10.1161/STROKEAHA.106.480848 17717319

[103] Tang WK Chen YK Lu JY Mok VCT Xiang YT Ungvari GS Microbleeds and post-stroke emotional lability. J Neurol Neurosurg Psychiatry (2009) 80:1082–6 10.1136/jnnp.2009.175372 19541687

[104] Fan YH Zhang L Lam WWM Mok VCT Wong KS Cerebral microbleeds as a risk factor for subsequent intracerebral hemorrhages among patients with acute ischemic stroke. Stroke (2003) 34:2459–62 10.1161/01.STR.0000090841.90286.81 12958325

[105] Roob G Lechner A Schmidt R Flooh E Hartung HP Fazekas F Frequency and location of microbleeds in patients with primary intracerebral hemorrhage. Stroke (2000) 31:2665–9 10.1161/01.STR.31.11.2665 11062292

[106] Yamada SS Periventricular and deep white matter leukoaraiosis have a closer association with cerebral microbleeds than age. Eur J Neurol (2012) 19:98–104 10.1111/j.1468-1331.2011.03451.x 21645176

[107] Schonewille WJ Singer MB Atlas SW Tuhrim S The prevalence of microhemorrhage on gradient-echo magnetic resonance imaging in acute lacunar infarction. J Stroke Cerebrovasc Dis (2005) 14:141–4 10.1016/j.jstrokecerebrovasdis.2005.05.003 17904015

[108] Haller S Bartsch A Nguyen D Rodriguez C Emch J Gold G Cerebral microhemorrhage and iron deposition in mild cognitive impairment: susceptibility-weighted MR imaging assessment. Radiology (2010) [cited 2012 Feb 17]. Available from: http://radiology.rsna.org.ezp.lib.unimelb.edu.au/content/early/2010/08/31/radiol.10100612.abstract 10.1148/radiol.10100612 20923870

[109] Cordonnier C van der Flier WM Sluimer JD Leys D Barkhof F Scheltens P Prevalence and severity of microbleeds in a memory clinic setting. Neurology (2006) 66:1356–60 10.1212/01.wnl.0000210535.20297.ae 16682667

[110] van den Boom R Lesnik Oberstein SAJ Ferrari MD Haan J van Buchem MA Cerebral autosomal dominant arteriopathy with subcortical infarcts and leukoencephalopathy: MR imaging findings at different ages – 3rd–6th decades. Radiology (2003) 229:683–90 10.1148/radiol.2293021354 14593195

[111] Viswanathan A Guichard J-P Gschwendtner A Buffon F Cumurcuic R Boutron C Blood pressure and haemoglobin A1c are associated with microhaemorrhage in CADASIL: a two-centre cohort study. Brain (2006) 129:2375–83 10.1093/brain/awl177 16844717

[112] Kapina V Vargas M-I Vulliemoz S Landis T Picard F Lalive PH VGKC antibody-associated encephalitis, microbleeds and progressive brain atrophy. J Neurol (2009) 257:466–8 10.1007/s00415-009-5370-5 19894073

[113] Hasiloglu ZI Albayram S Selcuk H Ceyhan E Delil S Arkan B Cerebral microhemorrhages detected by susceptibility-weighted imaging in amateur boxers. AJNR Am J Neuroradiol (2011) 32:99–102 10.3174/ajnr.A2250 20966064 PMC7964954

[114] Chen YW Gurol ME Rosand J Viswanathan A Rakich SM Groover TR Progression of white matter lesions and hemorrhages in cerebral amyloid angiopathy. Neurology (2006) 67:83–7 10.1212/01.wnl.0000223613.57229.24 16832082 PMC1502246

[115] Goos JDC Henneman WJP Sluimer JD Vrenken H Sluimer IC Barkhof F Incidence of cerebral microbleeds. Neurology (2010) 74:1954–1960 10.1212/WNL.0b013e3181e396ea 20548041

[116] Gregoire SM Brown MM Kallis C Jäger HR Yousry TA Werring DJ MRI detection of new microbleeds in patients with ischemic stroke five-year cohort follow-up study. Stroke (2010) 41:184–6 10.1161/STROKEAHA.109.568469 19892991

[117] Greenberg SM O’Donnell HC Schaefer PW Kraft E MRI detection of new hemorrhages: potential marker of progression in cerebral amyloid angiopathy. Neurology (1999) 53:1135–1135 10.1212/WNL.53.5.1135 10496283

[118] Jia Z Mohammed W Qiu Y Hong X Shi H Hypertension increases the risk of cerebral microbleed in the territory of posterior cerebral artery: a study of the association of microbleeds categorized on a basis of vascular territories and cardiovascular risk factors. J Stroke Cerebrovasc Dis (2013). 10.1016/j.jstrokecerebrovasdis.2012.12.016 23434162

[119] Liu W Liu R Sun W Peng Q Zhang W Xu E Different impacts of blood pressure variability on the progression of cerebral microbleeds and white matter lesions. Stroke (2012) 43:2916–22 10.1161/STROKEAHA.112.658369 22949472

[120] Black RS Sperling RA Safirstein B Motter RN Pallay A Nichols A A single ascending dose study of bapineuzumab in patients with Alzheimer disease. Alzheimer Dis Assoc Disord (2010) 24:198–203 10.1097/WAD.0b013e3181c53b00 20505438 PMC3715117

[121] Salloway S Sperling R Gilman S Fox NC Blennow K Raskind M A phase 2 multiple ascending dose trial of bapineuzumab in mild to moderate Alzheimer disease. Neurology (2009) 73:2061–70 10.1212/WNL.0b013e3181c67808 19923550 PMC2790221

[122] Bard F Cannon C Barbour R Burke RL Games D Grajeda H Peripherally administered antibodies against amyloid beta-peptide enter the central nervous system and reduce pathology in a mouse model of Alzheimer disease. Nat Med (2000) 6:916–9 10.1038/78682 10932230

[123] Daniëls R Geurts JJG Bot JC Schonewille WJ van Oosten BW Steroid-responsive edema in CAA-related inflammation. J Neurol (2009) 256:285–6 10.1007/s00415-009-0136-7 19159066

[124] Eng JA Frosch MP Choi K Rebeck GW Greenberg SM Clinical manifestations of cerebral amyloid angiopathy-related inflammation. Ann Neurol (2004) 55:250–6 10.1002/ana.10810 14755729

[125] Wilcock DM Rojiani A Rosenthal A Subbarao S Freeman MJ Gordon MN Passive immunotherapy against Abeta in aged APP-transgenic mice reverses cognitive deficits and depletes parenchymal amyloid deposits in spite of increased vascular amyloid and microhemorrhage. J Neuroinflammation (2004) 1:24 10.1186/1742-2094-1-24 15588287 PMC539292

[126] Wilcock DM Jantzen PT Li Q Morgan D Gordon MN Amyloid-beta vaccination, but not nitro-nonsteroidal anti-inflammatory drug treatment, increases vascular amyloid and microhemorrhage while both reduce parenchymal amyloid. Neuroscience (2007) 144:950–60 10.1016/j.neuroscience.2006.10.020 17137722 PMC1857306

[127] Iliff JJ Wang M Liao Y Plogg BA Peng W Gundersen GA A paravascular pathway facilitates CSF flow through the brain parenchyma and the clearance of interstitial solutes, including amyloid β. Sci Transl Med (2012) 4:147ra111 10.1126/scitranslmed.3003748 22896675 PMC3551275

[128] Ostrowitzki S Deptula D Thurfjell L Barkhof F Bohrmann B Brooks DJ Mechanism of amyloid removal in patients with Alzheimer disease treated with gantenerumab. Arch Neurol (2012) 69:198–207 10.1001/archneurol.2011.1538 21987394

[129] Sperling RA Bronen R Greenberg S Sorensen G Salloway S Gass A Three cases of apparent Vasogenic Edema (VE) from a phase 2 clinical trial of the gamma secretase Inhibitor BMS-708163 in patients with mild-to-moderate AD. Alzheimers Dement (2011) 7(4):S377 10.1016/j.jalz.2011.05.1085

[130] Carlson C Estergard W Oh J Suhy J Jack CR Jr Siemers E Prevalence of asymptomatic vasogenic edema in pretreatment Alzheimer’s disease study cohorts from phase 3 trials of semagacestat and solanezumab. Alzheimers Dement (2011) 7:396–401 10.1016/j.jalz.2011.05.2353 21784350

[131] Rosidi NL Zhou J Pattanaik S Wang P Jin W Brophy M Cortical microhemorrhages cause local inflammation but do not trigger widespread dendrite degeneration. PLoS One (2011) 6:e26612 10.1371/journal.pone.0026612 22028924 PMC3197572

[132] van Norden AGW van den Berg HAC de Laat KF Gons RAR van Dijk EJ de Leeuw F-E Frontal and temporal microbleeds are related to cognitive function: the Radboud University Nijmegen Diffusion Tensor and Magnetic Resonance Cohort (RUN DMC) Study. Stroke (2011) 42:3382–6 10.1161/STROKEAHA.111.629634 21940975

[133] Patel B Lawrence AJ Chung AW Rich P Mackinnon AD Morris RG Cerebral microbleeds and cognition in patients with symptomatic small vessel disease. Stroke (2013) 44:356–61 10.1161/STROKEAHA.112.670216 23321452

[134] Qiu C Cotch MF Sigurdsson S Jonsson PV Jonsdottir MK Sveinbjrnsdottir S Cerebral microbleeds, retinopathy, and dementia. Neurology (2010) 75:2221–2228 10.1212/WNL.0b013e3182020349 21172845 PMC3013588

[135] Werring DJ Frazer DW Coward LJ Losseff NA Watt H Cipolotti L Cognitive dysfunction in patients with cerebral microbleeds on T2*-weighted gradient-echo MRI. Brain (2004) 127:2265–75 10.1093/brain/awh253 15282216

[136] Poels MMF Ikram MA van der Lugt A Hofman A Niessen WJ Krestin GP Cerebral microbleeds are associated with worse cognitive function: the Rotterdam Scan Study. Neurology (2012) 78:326–33 10.1212/WNL.0b013e3182452928 22262748

[137] Gregoire SM Scheffler G Jäger HR Yousry TA Brown MM Kallis C Strictly lobar microbleeds are associated with executive impairment in patients with ischemic stroke or transient ischemic attack. Stroke (2013) 44:1267–72 10.1161/STROKEAHA.111.000245 23482601

[138] Arvanitakis Z Leurgans SE Wang Z Wilson RS Bennett DA Schneider JA Cerebral amyloid angiopathy pathology and cognitive domains in older persons. Ann Neurol (2011) 69:320–7 10.1002/ana.22112 21387377 PMC3228518

[139] Tang WK Chen Y-K Lu J-Y Wong A Mok V Chu WCW Absence of cerebral microbleeds predicts reversion of vascular “cognitive impairment no dementia” in stroke. Int J Stroke (2011) 6:498–505 10.1111/j.1747-4949.2011.00682.x 22111793

[140] Nighoghossian N Hermier M Adeleine P Blanc-Lasserre K Derex L Honnorat J Old microbleeds are a potential risk factor for cerebral bleeding after ischemic stroke. Stroke (2002) 33:735–42 10.1161/hs0302.104615 11872897

[141] Jeerakathil T Wolf PA Beiser A Hald JK Au R Kase CS Cerebral microbleeds: prevalence and associations with cardiovascular risk factors in the Framingham study. Stroke (2004) 35:1831–5 10.1161/01.STR.0000131809.35202.1b 15155954

[142] Altmann-Schneider I Trompet S de Craen AJM van Es ACGM Jukema JW Stott DJ Cerebral microbleeds are predictive of mortality in the elderly. Stroke (2011). Available from: http://www.ncbi.nlm.nih.gov/pubmed/21233474 10.1161/STROKEAHA.110.595611 21233474

[143] Tsushima Y Tanizaki Y Aoki J Endo K MR detection of microhemorrhages in neurologically healthy adults. Neuroradiology (2002) 44:31–6 10.1007/s002340100649 11942497

[144] Cordonnier C Al-Shahi Salman R Wardlaw J Spontaneous brain microbleeds: systematic review, subgroup analyses and standards for study design and reporting. Brain (2007) 130:1988–2003 10.1093/brain/awl387 17322562

[145] Vergouwen MDI de Haan RJ Vermeulen M Roos YBWEM Statin treatment and the occurrence of hemorrhagic stroke in patients with a history of cerebrovascular disease. Stroke (2008) 39:497–502 10.1161/STROKEAHA.107.488791 18174491

[146] Amarenco P Labreuche J Lipid management in the prevention of stroke: review and updated meta-analysis of statins for stroke prevention. Lancet Neurol (2009) 8:453–63 10.1016/S1474-4422(09)70058-4 19375663

[147] Hackam DG Woodward M Newby LK Bhatt DL Shao M Smith EE Statins and intracerebral hemorrhage: collaborative systematic review and meta-analysis. Circulation (2011) 124:2233–42 10.1161/CIRCULATIONAHA.111.055269 22007076

[148] Bokura H Saika R Yamaguchi T Nagai A Oguro H Kobayashi S Microbleeds are associated with subsequent hemorrhagic and ischemic stroke in healthy elderly individuals. Stroke (2011) 42:1867–71 10.1161/STROKEAHA.110.601922 21597015

[149] Jeong S-W Jung K-H Chu K Bae H-J Lee S-H Roh J-K Clinical and radiologic differences between primary intracerebral hemorrhage with and without microbleeds on gradient-echo magnetic resonance images. Arch Neurol (2004) 61:905–9 10.1001/archneur.61.6.905 15210529

[150] Greenberg SM Nandigam RNK Delgado P Betensky RA Rosand J Viswanathan A Microbleeds versus macrobleeds: evidence for distinct entities. Stroke (2009) 40:2382–6 10.1161/STROKEAHA.109.548974 19443797 PMC2758289

[151] Boulanger J-M Coutts SB Eliasziw M Gagnon AJ Simon JE Subramaniam S Cerebral microhemorrhages predict new disabling or fatal strokes in patients with acute ischemic stroke or transient ischemic attack. Stroke (2006) 37:911–4 10.1161/01.STR.0000204237.66466.5f 16469961

[152] Imaizumi T Horita Y Hashimoto Y Niwa J Dotlike hemosiderin spots on T2*-weighted magnetic resonance imaging as a predictor of stroke recurrence: a prospective study. J Neurosurg (2004) 101:915–20 10.3171/jns.2004.101.6.0915 15597750

[153] Thijs V Lemmens R Schoofs C Görner A Van Damme P Schrooten M Microbleeds and the risk of recurrent stroke. Stroke (2010) 41:2005–9 10.1161/STROKEAHA.110.588020 20651265

[154] Huang P Chen C-H Lin W-C Lin R-T Khor G-T Liu C-K Clinical applications of susceptibility weighted imaging in patients with major stroke. J Neurol (2011) [cited 2012 Feb 7]. Available at: http://www.ncbi.nlm.nih.gov/pubmed/22186853 10.1007/s00415-011-6369-2 22186853

[155] Soo YOY Yang SR Lam WWM Wong A Fan YH Leung HHW Risk vs benefit of anti-thrombotic therapy in ischaemic stroke patients with cerebral microbleeds. J Neurol (2008) 255:1679–86 10.1007/s00415-008-0967-7 19156486

[156] Sueda Y Naka H Ohtsuki T Kono T Aoki S Ohshita T Positional relationship between recurrent intracerebral hemorrhage/lacunar infarction and previously detected microbleeds. Am J Neuroradiol (2010) 31:1498–503 10.3174/ajnr.A2100 20448017 PMC7966111

[157] Arima H Tzourio C Anderson C Woodward M Bousser M-G MacMahon S Effects of perindopril-based lowering of blood pressure on intracerebral hemorrhage related to amyloid angiopathy: the PROGRESS trial. Stroke (2010) 41:394–6 10.1161/STROKEAHA.109.563932 20044530

[158] Gregoire SM Jäger HR Yousry TA Kallis C Brown MM Werring DJ Brain microbleeds as a potential risk factor for antiplatelet-related intracerebral haemorrhage: hospital-based, case-control study. J Neurol Neurosurg Psychiatry (2010) 81:679–84 10.1136/jnnp.2009.198994 20522874

[159] Naka H Nomura E Kitamura J Imamura E Wakabayashi S Matsumoto M Antiplatelet therapy as a risk factor for microbleeds in intracerebral hemorrhage patients: analysis using specific antiplatelet agents. J Stroke Cerebrovasc Dis (2013) 22(6):834–40 10.1016/j.jstrokecerebrovasdis.2012.06.001 22784819

[160] Wong KS Mok V Lam WWM Kay R Tang A Chan YL Aspirin-associated intracerebral hemorrhage: clinical and radiologic features. Neurology (2000) 54:2298–301 10.1212/WNL.54.12.2298 10881256

[161] Ge L Niu G Han X Gao Y Wu Q Wu H Aspirin treatment increases the risk of cerebral microbleeds. Can J Neurol Sci (2011) 38:863–8 22030424 10.1017/s0317167100012440

[162] Vernooij MW Haag MDM van der Lugt A Hofman A Krestin GP Stricker BH Use of antithrombotic drugs and the presence of cerebral microbleeds: the Rotterdam Scan Study. Arch Neurol (2009) 66:714–20 10.1001/archneurol.2009.42 19364926

[163] Huang Y Cheng Y Wu J Li Y Xu E Hong Z Cilostazol as an alternative to aspirin after ischaemic stroke: a randomised, double-blind, pilot study. Lancet Neurol (2008) 7:494–9 10.1016/S1474-4422(08)70094-2 18456558

[164] Biffi A Halpin A Towfighi A Gilson A Busl K Rost N Aspirin and recurrent intracerebral hemorrhage in cerebral amyloid angiopathy. Neurology (2010) 75:693–698 10.1212/WNL.0b013e3181eee40f 20733144 PMC2931649

[165] Lovelock CE Cordonnier C Naka H Al-Shahi Salman R Sudlow CLM Sorimachi T Antithrombotic drug use, cerebral microbleeds, and intracerebral hemorrhage: a systematic review of published and unpublished studies. Stroke (2010) 41:1222–8 10.1161/STROKEAHA.109.572594 20431083

[166] Alexander JH Lopes RD Thomas L Alings M Atar D Aylward P Apixaban vs. warfarin with concomitant aspirin in patients with atrial fibrillation: insights from the ARISTOTLE trial. Eur Heart J (2013) 7(4):S377 10.1093/eurheartj/eht445 24144788

[167] Wallentin L Lopes RD Hanna M Thomas L Hellkamp A Nepal S Efficacy and safety of apixaban compared with warfarin at different levels of predicted international normalized ratio control for stroke prevention in atrial fibrillation. Circulation (2013) 127:2166–76 10.1161/CIRCULATIONAHA.112.142158 23640971

[168] Connolly SJ Ezekowitz MD Yusuf S Eikelboom J Oldgren J Parekh A Dabigatran versus warfarin in patients with atrial fibrillation. N Engl J Med (2009) 361:1139–51 10.1056/NEJMoa0905561 19717844

[169] Siegal DM Crowther MA Acute management of bleeding in patients on novel oral anticoagulants. Eur Heart J (2013) 34:489–98 10.1093/eurheartj/ehs408 23220847

[170] Charidimou A Shakeshaft C Werring DJ Cerebral microbleeds on magnetic resonance imaging and anticoagulant-associated intracerebral hemorrhage risk. Front Neurol (2012) 3:133 10.3389/fneur.2012.00133 23015806 PMC3446731

[171] Hacke W Donnan G Fieschi C Kaste M von Kummer R Broderick JP Association of outcome with early stroke treatment: pooled analysis of ATLANTIS, ECASS, and NINDS rt-PA stroke trials. Lancet (2004) 363:768–74 10.1016/S0140-6736(04)15692-4 15016487

[172] Chalela JA Kang D-W Warach S Multiple cerebral microbleeds: MRI marker of a diffuse hemorrhage-prone state. J Neuroimaging (2004) 14:54–7 10.1177/1051228403258673 14748209

[173] Kidwell CS Saver JL Villablanca JP Duckwiler G Fredieu A Gough K Magnetic resonance imaging detection of microbleeds before thrombolysis. Stroke (2002) 33:95–8 10.1161/hs0102.101792 11779895

[174] Shoamanesh A Kwok CS Lim PA Benavente OR Postthrombolysis intracranial hemorrhage risk of cerebral microbleeds in acute stroke patients: a systematic review and meta-analysis. Int J Stroke (2012). 10.1111/j.1747-4949.2012.00869.x 22973896 PMC4067039

[175] Smith K Should cerebral microbleeds on magnetic resonance imaging contraindicate thrombolysis in patients with ischaemic stroke? A systematic review of the evidence. Radiography (2011) 17:254–9 10.1016/j.radi.2011.04.003

[176] Seo W-K Lee J-M Park MH Park KW Lee DH Cerebral microbleeds are independently associated with arterial stiffness in stroke patients. Cerebrovasc Dis (2008) 26(6):618–23 10.1159/000166837 18984946

[177] Lee S-H Park J-M Kwon S-J Kim H Kim Y-H Roh J-K Left ventricular hypertrophy is associated with cerebral microbleeds in hypertensive patients. Neurology (2004) 63:16–21 10.1212/01.WNL.0000132525.36804.A1 15249604

[178] de Laat KF van den Berg HAC van Norden AGW Gons RAR Olde Rikkert MGM de Leeuw F-E Microbleeds are independently related to gait disturbances in elderly individuals with cerebral small vessel disease. Stroke (2011) 42:494–7 10.1161/STROKEAHA.110.596122 21164137

[179] Greenberg SM Vonsattel JP Stakes JW Gruber M Finklestein SP The clinical spectrum of cerebral amyloid angiopathy: presentations without lobar hemorrhage. Neurology (1993) 43:2073–9 10.1212/WNL.43.10.2073 8413970

[180] Simonsen CZ Nielsen E Hypertensive microbleed as a transient ischemic attack mimic. Case Rep Neurol (2013) 5:31–3 10.1159/000348400 23525567 PMC3604869

[181] Watanabe A Kobashi T Lateral gaze disturbance due to cerebral microbleed in the medial lemniscus in the mid-pontine region: a case report. Neuroradiology (2005) 47:908–11 10.1007/s00234-005-1441-1 16142477

