# Supplementary material for: Cerebral Microbleeds: A Review of Clinical, Genetic, and Neuroimaging Associations
Source: Front Neurol. 2014 Jan 6;4:205. doi: 10.3389/fneur.2013.00205 (PMC3881231; doi:10.3389/fneur.2013.00205)
Supplement: Supplementary file 1 [file 73482_Yates_DataSheet1.ZIP › Supplementary Table 1.doc]

**Table 1: Microbleed Studies – Cross-sectional**

| **Author/year** | **Diagnosis** | **Prevalence** | **n** | **Age** | **Field Strength** | **Sequence** |
| --- | --- | --- | --- | --- | --- | --- |
| Jeerakathil et al., 2004 | Community-dwelling | 4.7% | 472 | 64.4 | 1.0T | T2*GRE |
| Qiu et al., 2010 | Community-dwelling | 12% | 3906 | 76.0 | 1.5T | T2*GRE |
| Poels et al., 2010 | Community-dwelling nondemented  Total  45-50y  50-59y  60-69y  70-79y  >80y | 15.3%  6.5%  11.5%  16.8%  28.9%  35.7% | 3979  412  1696  1350  377  143 | 60.3 | 1.5T | T2* GRE |
| Roob et al., 2000 | Community-dwelling | 6.4% | 280 | 59.9 | 1.5T | T2*GRE |
| Takashima et al., 2011 | Community-dwelling | 4% | 368 | 62.4 | 1.0T | T2*GRE |
| Yates et al., 2011 | Normal Controls | 19% | 84 | 74.6 | 3T | SWI |
| Gao et al., 2008 | Normal Controls | 8.7% | 23 | - | 3T | SWI |
| Haller et al., 2010 | Normal Controls | 14% | 35 | 63.7 | 3T | SWI |
| Yamada, 2012 | Normal Controls | 20% | 163 | 63.9 | 1.5T | T2*GRE |
| Tomofumi Nishikawa a et al., 2009 | Individuals without prior CVD  Total  40-49y  50-59y  60-69y  70-79y  >80y | 5%  12%  20%  20%  21%  17% | 698 | 64.5 | 1.5T | T2*GRE |
| Pettersen et al., 2008 | Normal Controls | 12% | 25 | 71.6 | 1.5T | T2*GRE |
| Tsushima et al., 2003 | Normal Controls | 3.7% | 1718 | 56.6 | 1.0T | T2*GRE |
| Kirsch et al., 2009 | Normal Controls | 0% | 33 | 71.2 | 1.5T | SWI |
| Cordonnier et al., 2006 | Subjective Memory Complainers | 10% | 184 | 61.0 | 1.0T | T2*GRE |
| Hanyu et al., 2003b | Normal Controls | 8% | 59 | 74.9 | 1.0T | T2*GRE |
| Chowdhury et al., 2011 | free from CVD | 2.3% | 1120 | 61.7 | 1.5T | T2*GRE |
| Boulanger et al., 2006 | IS/TIA | 19% | 236 | - | 3T | T2*GRE |
| Thijs et al., 2010 | IS/TIA | 25% | 487 | 72.0 | 1.0 or 1.5T | T2*GRE |
| Yamada, 2012 | ICH | 83% | 53 | 67.4 | 1.5T | T2*GRE |
| Tanaka et al., 1999 | ICH | 57% | 30 | 62.2 | 1.0T | T2*GRE |
| Tsushima et al., 2003 | ICH | 71% | 69 | 62.9 | 1.0T | T2*GRE |
| Jeon et al., 2007 | ICH | 68% | 220 | 58.3 | 1.5T | T2*GRE |
| Yakushiji et al., 2010 | ICH | 79% | 34 | - | 1.5T | T2*GRE |
| Orken et al., 2010 | ICH | 60% | 105 | 63.2 | 1.5T | T2*GRE |
| Alemany et al., 2006 | ICH | 64% | 45 | 65.0 | 1.5T | T2*GRE |
| Roob et al., 2000 | ICH | 54% | 109 | 64.6 | 1.5T | T2*GRE |
| Kato et al., 2002 | ICH | 71% | 35 | 62.9 | 1.5T | T2*GRE |
| Lee et al., 2011 | ICH | 57% | 20 | - | 1.5T | T2*GRE |
| Fiehler et al., 2007 | IS | 15% | 570 | 69.0 | NS | NS |
| Tang et al., 2009 | IS | 24% | 519 | 65.6 | 1.5T | T2*GRE |
| Yamada, 2012 | IS | 49% | 173 | 71.2 | 1.5T | T2*GRE |
| Tsushima et al., 2003 | IS | 18% | 232 | - | 1.0T | T2*GRE |
| Alemany et al., 2006 | IS | 18% | 45 | 68.0 | 1.5T | T2*GRE |
| Huang et al., 2011 | IS MCA territory | 32% | 44 | 71.9 | 3T | SWI |
| Fan et al., 2003 | IS | 35% | 43 | 68.0 | 1.5T | T2*GRE |
| Lee et al., 2011 | IS-LI | 38% | 60 | - | 1.5T | T2*GRE |
| Gao et al., 2008 | IS-LI | 26% | 35 | - | 3.0T | SWI |
| Fan et al., 2004 | IS-LI | 27% | 82 | 71.2 | 1.5T | T2*GRE |
| Yakushiji et al., 2010 | IS-LI | 36% | 22 | - | 1.5T | T2*GRE |
| Schonewille et al., 2005 | IS-LI | 46% | 46 | - | 1.5T | T2*GRE |
| Hanyu et al., 2003b | IS-LI multiple | 51% | 51 | 75.2 | 1.0T | T2*GRE |
| Kato et al., 2002 | IS-LI | 62% | 66 | 71.2 | 1.5T | T2*GRE |
| Lee et al., 2011 | IS-ATBI | 29% | 58 | - | 1.5T | T2*GRE |
| Kato et al., 2002 | IS-ATBI | 21% | 24 | 73.6 | 1.5T | T2*GRE |
| Gao et al., 2008 | IS-ATBI | 21% | 34 | - | 3.0T | SWI |
| Yakushiji et al., 2010 | IS-ATBI | 46% | 13 | - | 1.5T | T2*GRE |
| Lee et al., 2011 | IS-CEI | 28% | 33 | - | 1.5T | T2*GRE |
| Kato et al., 2002 | IS-CEI | 30% | 10 | 77.4 | 1.5T | T2*GRE |
| Gao et al., 2008 | IS-CEI | 4% | 24 | - | 3.0T | SWI |
| Yakushiji et al., 2010 | IS-CEI | 30% | 30 | - | 1.5T | T2*GRE |
| van den Boom et al., 2003 | CADASIL | 19% | 40 | 45 | 1.5T | T2*GRE |
| Viswanathan et al., 2006 | CADASIL | 35% | 147 | 51.8 | 1.5T | T2*GRE |
| Dichgans et al., 2002 | CADASIL | 69% | 16 | 49.5 | 1.5T | T2*GRE |
| Haller et al., 2010 | MCI  MCI-stable  MCI-progressive | 44%  35%  54% | 69  40  27 | 65.1  65.4  64.4 | 3T | SWI |
| Cordonnier et al., 2006 | MCI | 20% |  |  |  |  |
| Yates et al., 2011 | MCI | 36% | 28 | 75.9 | 3T | SWI |
| Kirsch et al., 2009 | MCI-stable | 4.3% | 23 | 75.5 | 1.5T | SWI |
| Kirsch et al., 2009 | MCI-progressive | 38% | 26 | 78.9 | 1.5T | SWI |
| Pettersen et al., 2008 | AD | 29% | 80 | 71.6 | 1.5T | T2*GRE |
| Hanyu et al., 2003a | AD | 32% | 59 | 77.3 | 1.5T | T2*GRE |
| Cordonnier et al., 2006 | AD | 18% | 223 | 70.0 | 1.0T | T2*GRE |
| Yates et al., 2011 | AD | 31% | 26 | 74.2 | 3T | SWI |
| Cordonnier et al., 2006 | VaD | 65% | 31 | 71.0 | 1.0T | T2*GRE |
| Hanyu et al., 2003b | VaD | 77% | 30 | 77.1 | 1.0T | T2*GRE |
| Won Seo et al., 2007 | VaD | 85% | 86 | 69.6 | NS | T2*GRE |
| Goos et al. | VaD | 82% | 11 | 70.6 | 3T | T2*GRE |
| van Rooden et al., 2009 | CAA-familial | 67% | 27 | 49.4 | 1.5T | T2*GRE |
