# Supplementary material for: Cerebral Microbleeds: A Review of Clinical, Genetic, and Neuroimaging Associations
Source: Front Neurol. 2014 Jan 6;4:205. doi: 10.3389/fneur.2013.00205 (PMC3881231; doi:10.3389/fneur.2013.00205)
Supplement: Supplementary file 1 [file 73482_Yates_DataSheet1.ZIP › Supplementary Table 2.doc]

**Table 2: Longitudinal Studies of Microbleeds**

|  | Study Design | | | | | | Microbleeds | | | Associations | | | |  |
| --- | --- | --- | --- | --- | --- | --- | --- | --- | --- | --- | --- | --- | --- | --- |
| Author | Population | n | Age | Duration | MB location specified | MRI Parameters | Baseline Prevalence | % Incident MB | Incidence (Rate) | Baseline MB | Incident MB | Vascular Events | Cognition | Other |
| Lee et al., 2011 | IS/TIA | 224 | 64.6 | 27 months | No | 1.5T T2*GRE | 34% | 23% | All ppt. 0.8/y  Baseline MB  0 MB 0.04/y  ≥1 MB 2.3/y  ≥2 MB 3.0/y  ≥5 MB 5.4/y |  | - Baseline WMH  - Baseline SVI  - Baseline ICH  - LDL-c |  |  | 11/76 had fewer MB at follow-up, associated with LDL-c |
| Poels et al., 2011 | Community | 831 | 68.5 | 3.4 years | Yes | 1.5T T2*GRE | 24% | 10.2% | - |  | All MB  - Age  - Baseline MB  - SBP, PP, HT  - APOE4E4  Lobar MB  - Age  - E4E4  - WMH volume  Deep MB  - Age  - SBP, HT  - Lacunar infarcts  - WMH volume |  |  | 6/831 had fewer MB at follow-up |
| Chen et al., 2006 | CAAH | 26 | 69.1 | 26 | No | 1.5T T2*GRE | - | 46% | - |  | - Baseline CMB  - WMH |  |  |  |
| Greenberg et al., 2004 | ICH | 94 | - | 28 months  34 with MRI at 16 months | Yes | 1.5T T2*GRE | - | 50% | - |  | - E2 and E4  - not age/sex/VRF | Baseline and Incident MB with  recurrent sICH | MB Incidence not associated with cognitive decline  Baseline Hemorrhage with subsequent cognitive impairment, functional dependence or death |  |
| Huang et al., 2011 | IS MCA | 44 | 71.9 | 7 days | No | 3T SWI | 32% | - | - |  | - | Baseline MB with subsequent hemorrhagic transformation |  |  |
| Tomofumi Nishikawa a et al., 2009 | No prior CVD | 698 | 65.4 | 3.5 years | No | 1.5T T2*GRE | 17% | - | - |  | - | Baseline MB with  - Stroke any  - IS  - Not ICH NS |  | HT also sig assoc with future CVD |
| Fan et al., 2003 | IS | 121 | 68.0 | 27 months | Yes | 1.5T T2*GRE | 35% | - | - | Higher DBP in Pt with Baseline MB  PHx ICH more common in MB patients |  | MB with ICH borderline  Baseline MB present in 5/6 patients with ICH and 5/11 with incident IS |  |  |
| Tsushima et al., 2003 | All-comers | 2019 139 | 56.6 | 468 days | Yes | 1.0T T2*GRE |  | - | - | Baseline MB with previous ICH |  | 4 pt with MB 2.9% had incident ICH |  |  |
| Bokura et al., 2011 | All-comers | 2102 | 62.1 | 3.6 years | Yes  52% deep, lobar 13%, mixed 34% | 1.5T T2*GRE | 4.4% | - | - | Baseline MB with  - age  - HT |  | MB with  - stroke any  - ICH. |  | ICH most common in deep/posterior structures  Younger demographic, cognitively normal. |
| Thijs et al., 2010 | IS/TIA | 487 | 72.0 | 2.2 years | Yes  Lobar 46%, Non-lobar 19%, mixed 35% | 1.0T 139  1.5T 244  3T 104  T2*GRE | 25.6% | - | - | Baseline MB with  - severe WMH  - PHx stroke |  | MB Overall  - no association with ICH, or recurrent IS.  Lobar/Mixed MB with Recurrent IS  No events in strictly deep MB |  |  |
| Imaizumi et al., 2004 | “SVD”  Deep ICH or lacunar IS | 337 | 66.0 | 22.5 months | Yes | 1.5T T2*GRE | - | - | - |  | - | Multiple Baseline MB subcortical independently associated with recurrent SVD =ICH and lacunar IS |  |  |
| Naka et al., 2006 | Stroke | 266 | 67.2 | 564.8 days | No | 1.0T T2*GRE | 35.3% | - | - |  | - | Baseline MB associated with subsequent ICH but not IS |  |  |
| Boulanger et al., 2006 | IS/TIA | 236 | - | 14 months | No | 3T T2*GRE or PW EPI | 19.1% | - | - | Baseline MB with  - Age  - DM  - WMH  - HT |  | Baseline MB independently associated with increased risk of diabling/fatal stroke, and death, but not all strokes |  |  |
| Nighoghossian et al., 2002 | IS | 100 | 60.0 | 8 weeks | Yes | 1.5T T2*GRE | 20% | - | - | Baseline MB with  - Age  - DM  - ATBI  - lacunae,  - antiplatelets |  | Baseline MB independently associated with CB after IS  Also assoc with CB were NIHSS score and DM |  |  |
| Jeon et al., 2007 | ICH | 63 | 58.4 | 23.3 months | No | 1.5T T2*GRE | 63% | 30.2% |  | Baseline MB with  - High SBP  - Low chol  - low LDL-c |  | Baseline MB with recurrent ICH |  |  |
| Sueda et al., 2010 | Recurrent ICH/IS | 55 | ICH 69.5  LI 72 | ICH 247.5 d  IS 179 d | Yes | 1.5T T2*GRE | - | - | - |  |  | Deep ICH more likely to correspond with site of prior MB than lobar ICH |  |  |
| Lee et al., 2008 | IS ATBI/CEI | 377 | 66.2 | 6 days | Yes | 1.5T T2*GRE | 29% | - | - |  |  | Baseline MB in 24% of HTf  MB not associated with HTf |  |  |
| Soo et al., 2008  Related to Fan e t al | IS | 908 | 68.4 | 27 months | Yes  Deep 40%  Lobar 18%  Mixed 35%  Infratentorial 7% | 1.5T T2*GRE | 28% | - | - |  |  | Baseline MB with ICH  Also  - age  - cortico-subcortical distribution  Mortality from ICH significantly higher with more baseline MB |  |  |
| Fiehler et al., 2007 | IS with TPA, multicentre | 570 | 69 | 1-10 days | No | Not stated, T2* | 15%  ≥5 MB 1.1% |  |  |  |  | SICH occurred in 5.8% with CMB and 2.7% without CMB p=0.17.  OR for SICH in CMB was 2.23 0.6-7.0 | -Lower prevalence of MB at baseline  -No multivariate analysis  -Not stratified by location  -Power calculation – n needed >1000, underpowered?Vernooij et al., 2008 |  |
| Kidwell et al., 2002 | IS with intra-arterial thrombolysis | 41 | - | 24 hours | No | 1.5T T2*GRE or EPI-SWI | 12% |  |  |  |  | Case Series  NS difference in prevalence of HT between MB+/-  P=0.049 for HT prevalence between multiple vs none/single MB  One pt had HT at site of prior MB, rest had HT at site of stroke |  |  |
| Chalela et al., 2004 | IS with TPA/Antiplt | 3 | - | 7 days | Yes | 1.5T T2*GRE | all | - | - |  |  | Case series: ICH post TPA/antiplatelet at site remote from MB or initial stroke |  |  |
